# Effective Drug Concentration and Selectivity Depends on Fraction of Primitive Cells

**DOI:** 10.3390/ijms22094931

**Published:** 2021-05-06

**Authors:** Jan Jakub Lica, Miłosz Wieczór, Grzegorz Jan Grabe, Mateusz Heldt, Marta Jancz, Majus Misiak, Katarzyna Gucwa, Wioletta Brankiewicz, Natalia Maciejewska, Anna Stupak, Maciej Bagiński, Krzysztof Rolka, Andrzej Hellmann, Andrzej Składanowski

**Affiliations:** 1 Department of Molecular Biochemistry, Faculty of Chemistry, University of Gdansk, 80-308 Gdansk, Poland; katarzynach3@wp.pl (K.G.); krzysztof.rolka@ug.edu.pl (K.R.); 2 Department of Physical Chemistry, Faculty of Chemistry, Gdansk University of Technology, 80-233 Gdansk, Poland; milafternoon@gmail.com; 3 Department of Microbiology, Harvard Medical School, 4 Blackfan Circle, Boston, MA 02115, USA; Grzegorz_Grabe@hms.harvard.edu; 4 Department of Pharmaceutical Technology and Biochemistry, Faculty of Chemistry, Gdansk University of Technology, 80-233 Gdansk, Poland; matheldt@student.pg.edu.pl (M.H.); marta.jancz@gmail.com (M.J.); majusmisiak@gmail.com (M.M.); wbrankiewicz@gmail.com (W.B.); nat.maciejewska@gmail.com (N.M.); chemmbag@pg.edu.pl (M.B.); askladan@pg.gda.pl (A.S.); 5 Polpharma Biologics S.A., Gdansk Science & Technology Park, Building A, 80-172 Gdansk, Poland; stupak.anna@gmail.com; 6 Department of Hematology and Transplantology, Medical University of Gdansk, 80-214 Gdansk, Poland; andrzej.hellmann@gumed.edu.pl

**Keywords:** cell culture density-dependent cytological stage profile, primitive acute myeloid leukemia cellular stages, primitive cancer cellular stages, drug screening platform, effective drug concentration, selectivity index, C-123

## Abstract

Poor efficiency of chemotherapeutics in the eradication of Cancer Stem Cells (CSCs) has been driving the search for more active and specific compounds. In this work, we show how cell density-dependent stage culture profiles can be used in drug development workflows to achieve more robust drug activity (IC_50_ and EC_50_) results. Using flow cytometry and light microscopy, we characterized the cytological stage profiles of the HL-60-, A-549-, and HEK-293-derived sublines with a focus on their primitive cell content. We then used a range of cytotoxic substances—C-123, bortezomib, idarubicin, C-1305, doxorubicin, DMSO, and ethanol—to highlight typical density-related issues accompanying drug activity determination. We also showed that drug EC_50_ and selectivity indices normalized to primitive cell content are more accurate activity measurements. We tested our approach by calculating the corrected selectivity index of a novel chemotherapeutic candidate, C-123. Overall, our study highlights the usefulness of accounting for primitive cell fractions in the assessment of drug efficiency.

## 1. Introduction

The stem cell theory of cancer states that some cancerous cells proliferate and sustain cell population similarly to stem cells in healthy organs and tissues. The idea that cancer is primarily driven by a small population of stem cells has important implications [1,2].

**Figure 1:**
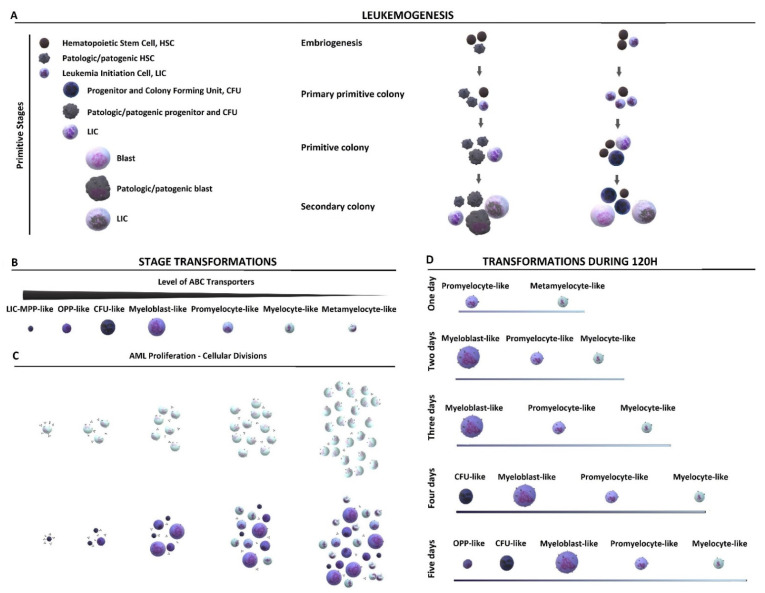
AML stage transformations. (**A**) Scheme of leukemogenesis and initiation of acute myeloid leukemia (AML). Left panel: Initiation of AML caused by the accumulation of DNA malfunctions resulting in the formation of LIC from the pathogenic primitive stage. LIC formation can occur independently in different cells and at different stages of their stage development. Right panel: Initiation of AML by LIC following the sudden and spontaneous appearance of multiple mutations in the primitive stage. Currently, LIC formation at a stage other than LSC HSC-like remains speculative. (**B**,**C**) Pattern of AML development and the effect of stage differentiation on interactions with a biologically active substance: (**B**) The surface level of ABC transporters decreases with the degree of differentiation. (**C**) As cells divide, the number of drug molecules per cell decreases. Top: hypothetical model of symmetrical divisions without maturation of the myelocyte-like stage. The model assumes that the “colony” would only be able to increase in the number of cells of the same stage: Myelocytes-like cells undergoing cell division terminally and maturing into metamyelocytes-like cells, unable to divide further and beginning to age. Bottom: Model of specific developmental stages of cell division with maturation, with a dynamic evolution of cytological stage fractions. (**D**) The stage transformations of AML over 5 days. The panel shows the AML stages capable of completing cell division within 120 h.

Acute Myeloid Leukemia (AML), one of the most studied and best understood malignancies, has well-described cellular stages. Despite numerous malfunctions at the cellular level, the hierarchical development of AML with retained ability to differentiate still resembles the hematopoietic process [3,4]. The AML leukemogenesis and its initiation by a Leukemia Initiation Cells (LICs) can occur either during embryogenesis or later in life, e.g., due to the accumulation of pathogenic malfunctions in Hematopoietic Stem Cells (HSCs) or degeneration of HSCs induced by the niche endothelial cells [5,6,7] (Figure 1A).

Once generated, the Leukemia Stem Cell HSC-like stage (LSC HSC-like) can directly transform into a LIC [5,6,7]. When the LIC originates from the Primitive Cell Stage (PC) other than LSC HSC-like cells (progenitor-like/Colony Forming Unit-like (CFU)/blast-like cell) that transforms into the LIC has to increase its own proliferative potential [8,9]. In such cases, the resulting leukemic cells are often associated with a Multi-Drug Resistance (MDR) phenotype, a poor prognostic for AML treatment [10,11,12,13,14]. MDR is prevalent in PCs due to stage-specific changes in the expression levels of ATP-Binding Cassette (ABC) transporters responsible for intra-/extracellular transport [10,11,15,16,17]. As a result, chemotherapeutics with high affinity for ABC transporters cannot achieve their desired pharmacological effect at concentrations applied in therapy [18]. The reduced effectiveness of conventional cytostatic agents is further affected by stage-dependent differences in the single-cell proliferation rate. One protein controlling these cellular stages is topoisomerase IIα [19,20,21,22]. Therapies based on functional inhibition of topoisomerase IIα are commonly used to eliminate fast-dividing maturing (blast-like, promyelocyte-like, myelocyte-like) AML cells [23]. However, this treatment often fails since PCs express low topoisomerase IIα levels [24]. This complex dynamic of protein expression patterns not only explains the deadly resilience of leukemic but also highlights the challenge in the robust and replicable quantification of drugs’ efficacies.

The increase of PC fraction over time, as well as their high differentiation potential, are attributed to a feedback mechanism observed in the late 1960s [25]. It was found that the secretion of cellular factors controls stage differentiation in vitro, an effect successfully used in the development of improved laboratory cell culture methodology [26,27,28,29,30]. Accordingly, in vitro cultivation of healthy PCs (including HSC) at low cell density enriches the PC fraction in culture, a process useful, e.g., in bone marrow transplant procedures [31]. Crucially, low cell density makes it possible to maintain high proliferation potential of human synovium-derived stem cell cultures [32]. As previously shown by our group, keeping HL-60 at low cell density by frequent passages significantly increased the PC content, including the LSC HSC-like stages [33]. Culturing at different specified cell densities resulted in three HL-60 sublines with various PC content, named Primitive, Standard, and Mature [33]. The establishment and cytological profiling of HL-60 sublines then served as a useful tool for determining the pharmacological parameters of compounds active against PCs [33].

Recently, it has become evident that many experiments and assays have been significantly affected by stage-dependent differences that are nontrivial to account for [33,34], as notoriously observed in the complex problem of the reproducibility of in vitro assays using cell models [35]. Even assuming that two laboratories remove all sources of error (such as different laboratory skills, types and volumes of multi-well plates, incubation times, cell density and viability, quality of culture media, equipment accuracies), the commonly used determination of the dose of a substance as the molar concentrations in the medium still has significant drawbacks [35,36,37]. Such a way of presenting results only informs about the initial extracellular concentration of the substance under study [36].

In a recently proposed alternative approach, the effective concentration of a compound that binds strongly or covalently to a defined molecular target is expressed as the number of molecules per cell [36]. The proposed model assumes that the desired cellular effect is obtained through the binding of a certain number of ligands to the molecular targets in a diffusion-controlled manner [36]. Despite being an interesting alternative, this model is not adequate for compounds with weak interactions with multiple molecular targets or acting as co-solvents. It also does not apply to cell stage-specific responses to the compound, and therefore does not fully solve the dosing problem of in vitro tests.

In this work, we show that the abovementioned problems can be addressed using screening platforms based on cell density-dependent stage profiles. Taking into account cellular stage is a simple improvement of the existing methods used in preclinical selection of PC-specific compounds. Accordingly, resistance indices of PCs should express the effective concentration as the number of drug molecules per primitive cell to directly compare the eradication potential of the tested substance. Applying this correction into screening platforms based on cell lines derived from leukemic and cancer patients should facilitate selection the optimal chemotherapeutic agent in personalized therapy, especially in the case of relapse.

### Highlights

We show that cell sublines with different stage composition can be derived from the HL-60, A-549, and HEK-293 human cell lines by manipulating cell culture density, highlighting the need for consistent culture conditions across different experiments. Here, we expand on previously described the HL-60 cell line stage profiles [33] and describe them more accurately using factors such as cytological morphology data, the expression of topoisomerase II, the presence of ABC transporters, viability and proliferative potential. Similarly, we characterize the cytological stage profiles of the A-549 cell line and its sublines based on the analysis of stage-specific morphology, DNA distribution, extracellular phosphatidylserine, ROS profiles. For HEK-293, we established three sublines based on morphology and ROS profiles.

In the HL-60 sublines, we calculated inhibitory concentrations (IC_50_) as well as effective concentrations (EC_50_, mol per cell and *EC_50_, mol per primitive cell) and selectivity indices for substances interacting with defined molecular targets (idarubicin and C-123) and substances with broad and nonspecific mechanisms of action (DMSO and ethanol). For the A-549 sublines, we determined the antiproliferative activity and selectivity indices of selected drugs and lead compounds with different mechanisms of action: C-123 (active against PCs in HL-60 and not affected by ABC transporters), C-1305 and doxorubicin (both specific topoisomerase IIα poisons), and bortezomib (proteasome inhibitor active against PCs in HL-60).

In our assays, we used a novel drug candidate, anthrapyridazone C-123 (Supplementary Text ST1), to demonstrate how the cell stage-based platform can inform us about the molecular mechanism of drugs active against CSCs. To improve characterize its mechanism of action, we investigated the induction of DNA breaks in the comet assay, induction of cellular senescence, generation of double-stranded DNA breaks, BrdU incorporation, kDNA decatenation, cytoskeleton interaction, in vitro proteasome inhibition, and in silico interactions with DNA.

Overall, we show how a multifactorial drug parameter determination of cell cytotoxicity with emphasis on primitive cell content can be used in the selection and identification of potent cancer drug development.

## 2. Results and Discussion

### 2.1. Cytological Culture Stages Profiles

Since the HL-60 line of AML cells is well characterized in terms of developmental stage profiles, we chose it as a reference line to study the stage-dependent quantitative aspects of drug efficiency. The other lines used in this study (non-AML cancerous A-549 and noncancerous HEK-293) serve, on the one hand, to provide a reference for the study of selectivity and, on the other hand, to extend the discussion of stage profiles beyond leukemic cells.

#### 2.1.1. HL-60

Lica et al. proposed a simple method for the determination of HL-60 stage profiles based on microscopic study (May-Grunwald-Giemsa staining of cells) and Side and Forward Scattering (SSC/FSC) cytometric analysis [33]. In that contribution, the profiles were supplemented by the study of stage-specific immunophenotype, DNA distribution, ROS generation, the induction of apoptosis, the intracellular level of glutathione (free radical scavenger), and the expression levels of genes responsible for the regulation of oxy-redox processes [33]. Morphological and physiological characteristics correlated well with the stage-dependent cellular response to the tested compounds, including clinically used chemotherapeutics with a well-established mechanism of action [33]. To make later discussion more generalizable, here we additionally study several functional characteristics of the sublines that are likely to contribute to the inter-subline differences in sensitivity to cytotoxic substances, as presented in Table 1. For detailed cytological characterization of HL-60 sublines see also Supplementary Text ST2 and Supplementary Figure S1.

**Figure 2:**
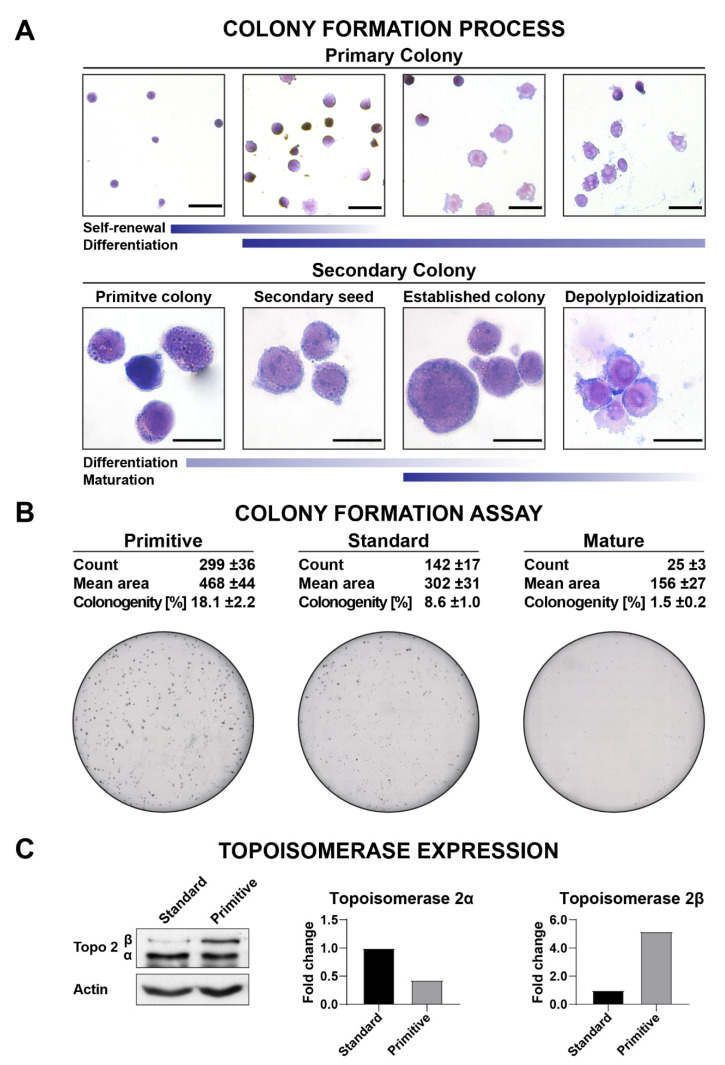
Primitive stages in HL-60. (**A**) Colony morphology. Micrographs show MGG-stained cells. Top panel: ×400 magnification. Bottom panel: ×1000 magnification. Marker: –20 µm. (**B**) Colony formation assay. Microphotographs show colonies formed by culture sublines. The values are averages ±SD of *n* = 3 independent experiments. (**C**) Expression of topoisomerase IIα isoforms in the HL-60 Standard and Primitive sublines.

Primitive HL-60. This subline is morphologically composed mostly from LSC HSC-like stages characterized by very low diameter (9–12 μm) and granularity, highly condensed chromatin, a nucleus with a diameter of 8–9 μm, as well as highly basis cytoplasm to blast-like stages with a larger diameter (20–50 μm), low granularity, immature chromatin, and a nucleus with few nucleoli (Figure 2A). As seen in Supplementary Figure S1A, the small and extensively proliferating HL-60 cells stained with JC-1 and hoechst 33,342 efficiently removed hoechst 33,342 from their interior in Supplementary Figure S1B, indicating that HL-60 primitive cells retain higher expression of ABC transporters, in accordance with the hierarchical morphological classification (Figure 1B). In the colony-forming assay (Figure 2B), Primitive HL-60 exhibited the highest clonogenicity, as reflected in the high percentage of PCs, especially with LSC/OPP/CFU-like stages. As shown in Figure 2C, Primitive HL-60 expressed topoisomerase IIα at a relatively lower level (two times) than Standard HL-60, suggesting lower susceptibility to topoisomerase IIα poisons. Overall, primitive stages accounted for ca. 85% of cells in the Primitive subline (Table 1).

Standard HL-60. This subline morphologically accounted for 90% of the maturating cellular stages, the blast-, promyelocyte- and myelocyte-like stages (Table 1). Promyelocyte- and myelocyte-like stages have highly granular, fine/more mature chromatin, and nucleus with (promyelocyte-like) or without nucleoli. Their average diameter is around 16–19 μm and cytoplasm is weakly basic to neutral. The clonogenicity of Standard HL-60 (Figure 2B) was only half of that observed for Primitive HL-60, reflecting the roughly halved population of primitive stages (ca. 40% compared to 85% and 18% compared to 9% for LSC/OPP/CFU-like stages). The transition to Standard HL-60 coincided with a dramatically decreased expression of the topoisomerase IIβ isoform (Figure 2C).

Mature HL-60. Due to the abundance of terminal stages of differentiation, this subline contains cells of many different shapes and sizes (see Figure 1, Supplementary Figures S1A and S2A). Slowly proliferating or nondividing cells (Supplementary Figure S1B) were less capable of removing hoechst 33,342 from their interior. The clonogenicity of Mature HL-60 (Figure 2B) was reduced ca. 12-fold with respect to Primitive HL-60, reflecting a dramatic drop in the fraction of primitive stages (ca. 4% compared to 85% in Primitive HL-60) and including self-renewal and differentiation stages (18% compared to 1.5% in Primitive HL-60).

#### 2.1.2. A-549 Cell-Density Cytological Stage Profile

As in case of HL-60, tight control of the cell density enabled us to establish three sublines, named Primitive, Standard, and Mature, in the A-549 cell line. The obtained cytological stage profiles are briefly characterized in Table 2.

**Figure 3:**
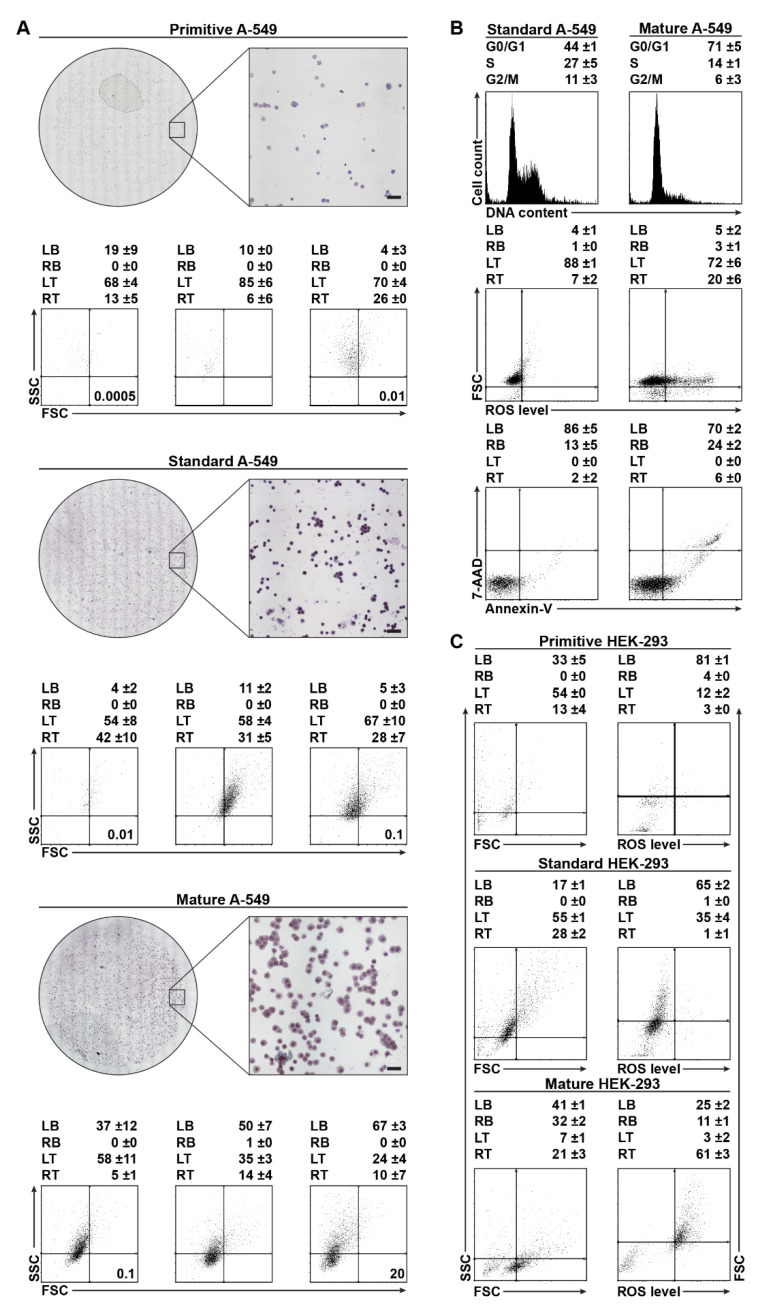
Cytological stage profiles derived at different cell culture densities. (**A**) Morphology of the A-549 subline. Micrographs of MGG-stained cells and SSC/FSC cytometric parameters. Marker: –50 µm. (**B**) Cell cycle progression, ROS generation, and extracellular phosphatidylserine in the A-549 Standard and Mature sublines. (**C**) HEK-293 morphology (laser light scattering) and ROS effect. All values are averages ±SD of *n* = 3 independent experiments. LB—left bottom, RB—right bottom, LT—left top, RT—right top.

Primitive A-549. The A-549 cell line cultured at cell densities between 0.25 and 2.5 × 10^3^ cells per 175 cm^2^ is formed mainly by cells of small size (9–11 µm) characterized by highly condensed chromatin, high nucleus-to-cytoplasm ratio (0.9–0.6), and highly alkaline cytoplasm (Figure 3A). On the SSC/FSC scatterplot, up to 90% of these cells are located in the left top (LT) square, forming two weakly separated fractions (Figure 3A).

Standard A-549. The A-549 cell line grown at cell densities from 2.5 to 50 × 10^3^ cells per 175 cm^2^ is initially formed by small cells located in the LT (about 60%) and right top (RT) (about 40%) (Figure 3A). At a density of 50 × 10^3^ cells per 175 cm^2^, the fraction of cells located in the RT increased up to 75% (Figure 3A). Increased RT fraction with a simultaneous decrease in left bottom (LB) square correlated with an increase in the number of large cells ranging in size from 11 µm to 15 µm, as well as with lower chromatin condensation, less alkaline cytoplasm, and lower nucleus to cytoplasm ratio (0.6–0.2; Figure 3A). About half of the cells were in the G_0_/G_1_ cell cycle phase, and about four-times less were in the G_2_/M phase (Figure 3B). Based on the population doubling time and low percentage of ROS-positive cells, it can be assumed that these cell cycle values correspond to a proliferative population. The fraction of aging/senescent (nondividing) cellular stages was usually less than 10% (Figure 3A,B).

Mature A-549. The A-549 cell line grown at densities higher than 50 × 10^3^ cells per 175 cm^2^ resulted in ca. 70% of the population located in LT and LB of the SSC/FSC dot-plot (Figure 3A). Growth of cells corresponding to the LB square was reflected in the change of the population profile (Figure 3A), leading to an increased number of bigger (15–25 µm) cells, a slightly acidic cytoplasm, and lower nuclear/cytoplasm ratio (0.2–0.05) (Figure 3A,B). Compared to Standard A-549, the number of cells in the G_0_/G_1_ cell cycle phase increased, while the number of cells in the G_2_/M phase decreased by about 50% (Figure 3B), meaning that about 50% of cells did not undergo further cell division (Figure 3B). Cytometric analysis of intracellular ROS level and extracellular presentation of phosphatidylserine showed that about 30% of the population was ROS-positive, with a tendency toward cell death (Figure 3A,B).

#### 2.1.3. HEK-293 Morphology and ROS

It has been shown that under the influence of biological manipulations, HEK-293 cells are able to form cytologically, immunophenotypically, proteomically, and genetically different culture sublines with the ability to form colonies in soft agar [59,60]. We thus cultured this line at different cell densities to derive and analogously characterize the three sublines to HL-60 and A-549.

Primitive HEK-293. The cells cultured at cell densities from 0.25 to 10 × 10^3^ cells per 175 cm^2^ are located in LB and LT squares (about 90% of the population) and in the RT square (Figure 3C) of the FSC/SSC scatterplot.

Standard HEK-293. Among the cells cultivated at cell densities from 10 to 100 × 10^3^ cells per 175 cm^2^, the percentage of cells localized on the FSC/SSC scatterplot in LB is halved. In the LT, the number of cells does not change significantly. However, in the RT, the number of cells is ca. 3-fold higher compared to Primitive HEK-293 (Figure 3C).

Mature HEK-293. The subline obtained at cell densities above 100 × 10^3^ cells per 175 cm^2^ consisted of 60% of ROS-positive cells located on the FSC/SSC scatterplot, mainly in the RT square and partially in the LB squares (Figure 3C). The ROS-positive Mature HEK-293 cells, located in LB, had lower SSC and higher FSC parameters than the ROS-negative cellular stages located primary in this square (Figure 3C).

### 2.2. Effective Concentration as Molecules per (Primitive) Cell

Due to the multifactorial and complex nature of the problem of dosing in cell models, drug effectiveness should optimally be presented using several measures of activity. However, such multicomponent reporting degrades the readability of the results and complicates direct comparison between drug candidates.

We analyzed and built on the concept of an effective concentration unit (EC_50_), defined as IC_50_ normalized to the initial quantity of the total cell number. The fraction of primitive cells improved the presentation of results and made the biological activity data more informative.

We observed that the differences in IC_50_ and EC_50_ revealed drug cytotoxicity specificities. For example, values of IC_50_ and EC_50_ independent of the initial total cell number are indicative of a solvent-like behavior (ethanol), values of per-primitive cell EC_50_—denoted here as *EC_50_ (as well as *IC_50_)—that remain constant independent of the initial primitive cell density strongly suggest that the compound is effective against primitive cells. Below, we confront the known stage-specific morphological and functional features with the drugs’ mechanisms of actions to determine whether the two descriptions—macroscopic (effective concentrations) and microscopic (molecular targets)—complement each other.

#### 2.2.1. Mechanism of Action of tested Drugs

To enable direct confrontation of the above results with the known mechanisms of actions of the individual drugs used in this study, we compiled the current mechanistic knowledge on them in Table 3. Results and discussion on their basal mechanism of action can be found in Supplementary Figure S2, Supplementary Table S1, and Supplementary Text ST1, ST3 (using HL-60 as a reference), as well as in Supplementary Figures S3 and S4, Supplementary Table S1, Supplementary Text ST1, ST4 and Supplementary Video-Movies SVM3, SVM4 (using A-549).

Since C-123 is a novel drug candidate which was first described on a mechanistic level in our previous report [33,61], below we briefly justify the above findings regarding its broad efficiency in primitive cells. For a detailed description of its cytotoxic properties, see Supplementary Text ST1, ST3, ST4 and Supplementary Table S1.

**Figure 4:**
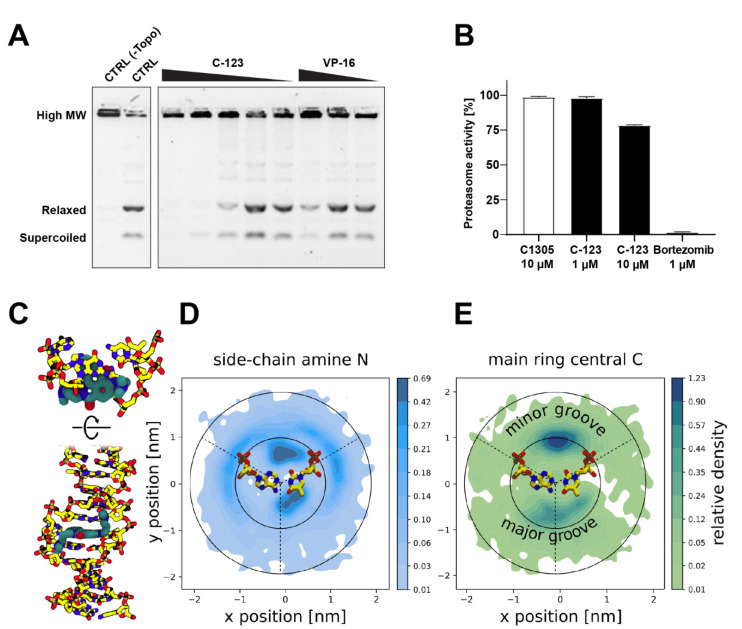
C-123 studies in vitro. Topoisomerase IIα and proteasome inhibition assays and molecular modeling of the interactions of C-123 with DNA. (**A**) Inhibition of topoisomerase IIα as measured by in vitro decatenation assay. (**B**) Quantification of proteasome inhibition by C-123 as measured by in vitro assay on A-549 cell lysates. Bortezomib was used as a positive control. The values represent averages of *n* = 3 independent experiments ±SEM. (**C**) Side and top views of an intercalated complex in which C-123 displaced an AT pair while interacting with the phosphosugar backbone via positively charged side chains. (**D**,**E**) Relative densities of the side-chain amino nitrogens of C-123 (**D**) or a central carbon from the fused ring core (**E**) integrated along the DNA axis, shown in a local coordinate system with the minor groove on the top and major groove on the bottom (see the schematic base pair for approximate location of bases and backbone). Note the nonlinear scale in the color bars.

C-123 is an anthracycline derivative (Supplementary Text ST1) that is particularly active against CSCs [33]. In in vitro assays, it showed a concentration-dependent ability to impair topoisomerase IIα functioning (Figure 4A), but this activity was moderate compared to other anthrapyridazones and could not be observed in cellular models (Supplementary Texts ST3 and ST4). Furthermore, in vitro proteasome inhibition (~25%) was achieved for C-123 at concentrations 10-fold higher than that of bortezomib sufficient for complete proteasomal shutdown (Figure 4B). This moderate inhibitory activity on proteasome might be one of the main mechanisms of C-123 specificity for PCs.

Additionally, C-123 has shown the ability to induce DNA single- or double-strand breaks independently of ROS production (Supplementary Figures S3, S4 and Supplementary Text ST3, ST4). For this reason, we performed equilibrium Molecular Dynamics simulations and analyzed the interactions of C-123 with double-stranded DNA, finding minor groove binding modes with long residence times (above 2 μs) and limited specificity for AT, with AT base pairs and phosphate groups being good hydrogen bond acceptors for the protonated tertiary amines of C-123 side chains (Supplementary Video Movie SVM1, Figure 4D,E). The artificially induced high-concentration conditions also allowed for the observation of a spontaneous intercalation event within 2 μs, suggesting that intercalation is a prominent interaction mode between C-123 and DNA (Supplementary Video Movie SVM2, Figure 4C). Thus, this event partially explains the broad activity against multiple types of cells, likely through the induction of senescence and/or cell death mediated by the DNA damage response (Supplementary Video Movie SVM3, Supplementary Figures S3 and S4, Supplementary Table S1). Interestingly, the formation of double-strand breaks is more prominent in HL-60 than A-549 (Supplementary Table S1), hinting at a yet unidentified cell type-specific mechanism of induction of strand breaks that would confer selectivity against leukemia cells.

#### 2.2.2. Total and Primitive Cell Dependence of IC_50_ in HL-60 Cells

**Figure 5:**
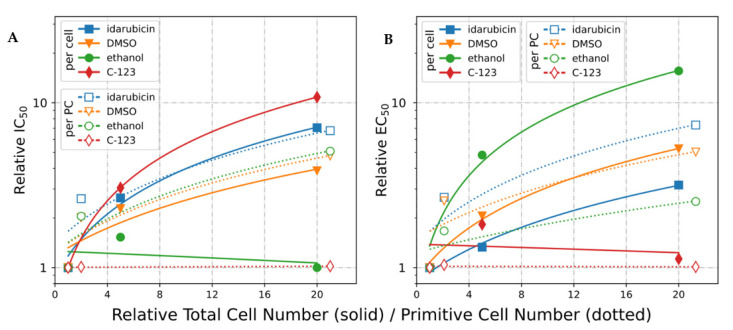
Relative IC_50_ and EC_50_ for C-123, idarubicin, ethanol, and DMSO as a function of the relative initial number of cells or primitive cells in HL-60, shown as linear fits to the data. (**A**) Relative IC_50_ as a function of relative total initial cell number (solid line) or total initial primitive cell number (dotted line). Solid line: IC_50_ values obtained at initial densities (5, 25, and 100 × 10^3^ cells per mL) were normalized relative to the value at lowest cell density (5 × 10^3^ cells per mL) and averaged over the 3 sublines: Primitive, Standard, and Mature. These averages were plotted as a function of multiples of the lowest cell density (1, 5, and 20 corresponding to 5, 25, and 100 × 10^3^ cells per mL). Dotted line: An analogous procedure was performed considering the fractions of primitive cells in each subline (0.04 in Mature, 0.4 in Standard, and 0.85 in Primitive; Table 1). *IC_50_ values were normalized to the Primitive subline, and the relative values were averaged over the 3 initial densities. The averaged values were plotted as a function of multiples of the lowest fraction of primitive cells (1, 2.1, and 21 corresponding to the Primitive/Primitive, Primitive/Standard, and Primitive/Mature ratios). (**B**) Normalization of EC_50_ as a function of relative initial cell number, calculated per total (solid line) or primitive (dotted line). Solid line: EC_50_ values obtained at initial densities (5, 25, and 100 × 10^3^ cells per mL) were averaged over the 3 sublines and normalized relative to the value at lowest cell density (5 × 10^3^ cells per mL). These averages were plotted as a function of multiples of the lowest cell density (1, 5, and 20 corresponding to 5, 25, and 100 × 10^3^ cells per mL). Dotted line: *EC_50_ values were averaged over the 3 initial densities and normalized to the Primitive subline. The normalized values were plotted as a function of multiples of the lowest fraction of primitive cells (1, 2.1, and 21 corresponding to the Primitive/Primitive, Primitive/Standard, and Primitive/Mature ratios). EC_50_ and *EC_50_ averaged values are presented in panel Table 4.

We performed the MTT cell proliferation assay for the three HL-60 sublines using drugs either with high affinity to their targets (idarubicin and C-123) or low-specificity cosolvents (DMSO and ethanol) where the variable was the initial number of cells used for plating (Table 4) [33,62,63]. The highest inhibitory activity (IC_50_, the concentration required to achieve 50% inhibition of culture proliferation) against HL-60 cells was measured for idarubicin and C-123 (Table 4). Interestingly, C-123, idarubicin, and DMSO displayed significant relative differences of calculated IC_50_ values dependent on initial cell densities. This dependence was not observed for ethanol (Table 4 and Figure 5A). In the case of C-123, the IC_50_ increased 3- and 11-fold for 5- and 20-fold higher initial cell densities, respectively. For idarubicin, the same IC_50_ for corresponding initial cell densities resulted in respective 2.5- and 7-fold IC_50_ increases. Last, for DMSO, the observed IC_50_ increased 2- and 4-fold (Table 4 and Figure 5A). The “drug-like” behavior with cell density dependence in the case of DMSO is consistent with its reported low-affinity interactions on specific cellular targets [64]. This contrasts with ethanol, whose activity depends on the overall concentration [65,66].

When the variable was the initial quantity of primitive cells (Figure 5A), we observed major changes for idarubicin, with DMSO and ethanol both following a very similar trend, but the respective values remained constant for C-123. For idarubicin, a 2-fold difference in the number of primitive cells translated into an approximately 3-fold difference in *IC_50_, while a 20-fold primitive cell difference resulted in a 7-fold increase in *IC_50_ (Figure 5A). For DMSO and ethanol, the respective *IC_50_ increases were approximately two- and five-fold.

Overall, the IC_50_ of C-123, idarubicin, and DMSO in HL-60 cells was dependent on the initial number of cells, while the IC_50_ of ethanol was not. In the case of drugs medium active against primitive cells such as idarubicin (targeting mainly maturing and senescent cells), and weakly active like DMSO, and ethanol, the *IC_50_ depended on fraction of these resistant cells. The IC_50_ and *IC_50_ trend lines are comparable, suggesting potential variable PC content during the experiment. For compounds active against primitive cells such as C-123, the increase of IC_50_ values was only observed with the total cell count, and not with the fraction of primitive cells (Figure 5A).

#### 2.2.3. IC_50_ Normalized to Cell Number (EC_50_)

The concept of IC_50_ normalization with respect to the number of cells, presented by Doskey et al. [36], achieves reasonably consistent values for compounds binding to a well-defined molecular target, such as idarubicin (Figure 5B, solid line). The EC_50_ values for DMSO diverged slightly more (5-fold) while, in the case of ethanol, the difference was 20-fold. As mentioned earlier, this effect can be either due to an interaction at the cosolvent level (entirely concentration-dependent) or due to density-dependent changes in cellular stage profiles that affect sensitivity to the given substance. Hence, we confirm the prediction by Doskey et al. that the cytotoxicity of compounds lacking a well-defined target will not be robustly quantified by their model [36]. Moreover, the fact that C-123 displayed stronger dependence than idarubicin and DMSO on the total cell number suggests that this drug has better pharmacological properties (e.g., specificity, affinity) than idarubicin and DMSO (Figure 5B, solid line).

The lack of consistent EC_50_ values in the studied compounds could be explained by temporal cell stage dynamics displaying variable tolerance to tested drugs. Higher cell densities (total cell number) result in a greater fraction of senescent cells that often undergo apoptosis. Cell death could lead to local drug concentration fluctuations and could affect the drug tolerance of neighboring cells. This scenario would be particularly relevant for cell sublines with high starting cell densities such as Mature HL-60.

Therefore, in order to precisely determine the EC_50_ for a culture containing substantial PC content, we recommend maintaining the primary phenotype during the experiment for as long as possible, ideally through culturing at low cell densities. The time of assay should also be taken into account as another variable important in making the stage-specific calculations robust and replicable (Figure 1C,D).

#### 2.2.4. IC_50_ Normalized to Primitive Cell Number (*EC_50_)

Despite a significant improvement in clarity and reproducibility, the model proposed by Doskey [36] does not account for the variability of cellular stages present in the culture. In our experiments, we found important differences in EC_50_ values of three compounds (idarubicin, DMSO, ethanol) between the studied sublines (Table 4).

The Mature HL-60 subline, rich in senescent cell forms, was the most sensitive to these tested drugs, while the PC-rich Primitive HL-60 subline was most resistant, suggesting that primitive cell content could be a major driver of these EC_50_ discrepancies (Table 4). Therefore, we propose a new pharmacokinetic measure, designated as *EC_50_, that normalizes the efficient drug concentration to the primitive cell content. By calculating *EC_50_ (EC_50_ per primitive cell instead of per cell), we significantly lowered variability in the results for drugs when multiple sublines were compared (Figure 5B), suggesting that ethanol, DMSO, and idarubicin are weakly/medium active against primitive cells.

Similar to relative normalization IC_50_, the normalized EC_50_ parameter was likely a result of the strength (the mean interaction force being the resultant of all effects on various cells and molecular targets in the population) of the interaction between the drug and its target (total cell normalization) or its specificity toward PC (PC normalization).

Obtained *EC50 trends provide an insight into the cytotoxicity mechanism of a studied drug and can inspire more targeted mechanistic studies crucial for the drug development pipeline. The stronger the interaction between the tested compound and its molecular target, the flatter the EC_50_ trend line. By the same token, the more PC-specific drug, the flatter *EC_50_ trend line.

In case of compounds moderately specific toward PC, the *EC_50_ was expected to increase with the fraction of primitive cells in the culture until 100% PC was reached. Using the 5 × 10^3^ cells per mL initial density that steadily maintained the Primitive HL-60 phenotype, the extrapolated per-primitive cell *EC_50_ (i.e., assuming from 85% to 100% primitive cells content/number) for idarubicin, DMSO and ethanol was 1.6 fmol per cell, 80 μmol per cell, and 121.8 μmol per cell respectively.

In contrast, for a compound equally active against primitive and nonprimitive cells (*EC_50_ is not affected by stage-dependent resistance) such as C-123, an almost constant value (less than two-fold difference) was achieved through per total cell normalization (Figure 5B). Per-primitive cell normalization further improved the consistency of EC_50_ (Figure 5B and Table 4).

In case of PC-active drugs such as C-123, the extrapolated per-primitive cell EC_50_ (i.e., assuming 100% primitive cells) trend line was stable and consistent. Even when the PC content increased substantially (e.g., the increase between Mature and Primitive HL-60 at seeding density 5 × 10^3^ cells per mL), the *EC_50_ value remained virtually the same for C‑123 (approximately 5% change) while, for idarubicin, the *EC_50_ was significantly higher (approximately 1400% change).

We note here that, in the case of drugs for which stage-specific resistance is observed (e.g., change in topoisomerase IIα, ABC transporter, and glutathione levels), the Primitive stage profile (and thus stage-dependent measures of cytotoxicity) will also drift and evolve over time. Due to the stage-specific expression patterns discussed above, the proliferating and highly drug-resistant non-maturing primitive (LSC HSC-like, OPP-like, CFU-like) cells will compensate for the loss of maturing primitive (blast-like) cells, eventually should leading to an increase in the observed values of IC_50_ and EC_50_.

#### 2.2.5. IC_50_ and EC_50_ Calculated Using A-549 Cells

Here, we performed the MTT proliferation assay for four compounds: C-123 and bortezomib on the derived Primitive Standard and Mature A-549 sublines (Figure 3A,B and Table 2), and C-1305 and doxorubicin on the derived Standard and Mature A-549 (Table 5), for the calculation of IC_50_. The constant cell number (0.25 × 10^3^ cells per well of a 96-well plate) of Primitive, Standard, and Mature A-549 is essential to calculate EC_50_.

All tested drugs showed biological activity IC_50_ in the micromolar range and EC_50_ in the femtomolar range. The greatest correlation between changes in the fractions of stage forms and the biological effect was demonstrated for the topoisomerase poisons C-1305 and doxorubicin (Table 5). Differently than in the case of idarubicin and HL-60, the Mature subline of A-549 was the least sensitive to both C-1305 and doxorubicin (Table 4 and Table 5). This lower sensitivity of Mature A-549 to topoisomerase IIα poisons is likely explained by a greater (up to two-fold) percentage of nondividing senescent stages (in total, about 85–90%) and a modest fraction of rapidly proliferating PC blasts (Figure 3A,B, Table 2). In the absence of topoisomerase IIα, other mechanisms of cytotoxicity of doxorubicin and C-1305 take over at higher concentrations. In the case of the proteasome inhibitor bortezomib, the IC_50_ and EC_50_ values (Table 5) of Primitive, Standard, and Mature A-549 are nearly identical, showing the effective elimination of PC, including CSCs. Similarly, C-123 showed efficacy in the elimination of CSCs, although at a three-fold higher concentration (Table 5).

The use of four substances (C-123, bortezomib, C-1305, doxorubicin) with different mechanisms of action and/or resistance patterns highlights the complexity of the dosing problem in in vitro cell models.

### 2.3. Selectivity Index Quantification

The selectivity index is frequently reported in the literature as a simple ratio of IC_50_ calculated for healthy and cancer cells [72,73,74,75], with values higher than 1 indicating desirable selectivity against cancer cells. Subsequently, these values are often compared across different cell types at different stages of development and different initial cell densities. As we show in this work, IC_50_ values are highly dependent on the initial numbers of both all cells and primitive cells. For the same reason, the selectivity index calculated in such a way is a very imprecise measure and will exhibit significant variability. Below, we show how the use of per-cell EC_50_ values in the calculation of selectivity indices can impact the conclusions regarding specific activity against individual sublines.

#### 2.3.1. HL-60

In the case of SI calculated based on EC_50_ (and IC_50_), the selectivity index should be compared with that obtained with use of *EC_50_ and *IC50 that can be calculated at the most primitive cell stage, preferably at initial cell densities below 5 × 10^3^ cells per mL for Primitive HL-60 and 0.5 × 5 × 10^3^ × 10^3^ cells per mL for HEK-293. When a tested substance is active toward PCs (bortezomib and C-123), the *EC_50_ parameter remains unchanged in sublines with various PC content.

Using the Primitive HL-60 subline with bortezomib (Table 6) and all three HL-60 sublines with C-123, we calculated the IC_50_- and EC_50_-based selectivity indices, comparing them with the healthy HEK-293 cells as a reference. In case of C-123, the IC_50_-based selectivity index changed from slightly favorable (>>1) to largely unfavorable (<1) with increasing initial cell densities, with a 15-fold difference between the lowest and highest density. As before, we saw no dependence on the subline (as proxy for number of primitive cells).

In contrast, using the per-cell normalized selectivity index that is based on EC_50_ values, we obtained values that are not only comparable but also much more favorable (>>1) than ones based on IC_50_. These values, ranging from 15 to 30, indicate that C-123 is indeed selective toward AML cells and provide a much more robust estimate than obtained the values based on IC_50_.

#### 2.3.2. A-549

None of the tested compounds (bortezomib, C-123, doxorubicin, C-1305) showed selective cytotoxicity against A-549 cells. The highest observed values slightly exceeded 1 for C-1305 and doxorubicin (Table 5), but the recalculation of selectivity indices based on EC_50_ yielded lower estimates of selectivity in virtually all cases (Table 5).

#### 2.3.3. Selectivity Index Summary

By comparing the IC_50_ and EC_50_ values obtained for A-549 to those obtained for healthy embryonic kidney cells HEK-293, we found that C-123, bortezomib, doxorubicin, and C-1305 all had selectivity indices lower than 1, indicating higher cytostatic activity in embryonic than cancer cells (Table 4, Table 5 and Table 6). However, despite the lack of clear-cut anticancer selectivity, C-123 turned out to be much less toxic for healthy HEK-293 cells than bortezomib, a drug used e.g., in multiple myeloma therapy (Table 5) [76,77].

In the HL-60 cells, the EC_50_-based analysis revealed that C-123 is a largely selective antileukemic cytotoxic agent, with a selectivity index of 15 (using the most reproducible conditions, i.e., Primitive subline at low densities 5 × 10^3^ cells per mL). On the other hand, this conclusion does not apply to bortezomib, for which the selective index was calculated at 0.34. As a result, we noted that C-123 was from 43-fold to 81-fold more selective than bortezomib against AML when selectivity indices were calculated on Primitive sublines using EC_50_.

## 3. Materials and Methods

### 3.1. Software, Equipment, and Statistical Analysis

Cell density was measured using Coulter Z2 (Beckman, Indianapolis, IN, USA) equipped with 100 mm aperture or using the flow cytometer Guava EasyCyte 8HT (Merck KGaA, Darmstadt, Germany). Microscopy images were acquired with Olympus BX60 epifluorescence microscope coupled to XC50 CCD camera and equipped with plan fluorite objectives (Olympus, Center Valley, PA, USA) (air objectives 20×, NA = 0.5, 40×, NA = 0.75 and oil (type F, *n* = 1.518) objective 60×, NA = 1.25). Microscopy images were acquired with Olympus BX60 epifluorescence microscope coupled to the XC50 CCD camera. Scans of the entire preparations were performed with the Olympus IX83-motorized microscope equipped with the plan fluorite objective (20×, NA = 0.45). Images were analyzed in CellSens Standard (Olympus, Tokio, Japan) or Fiji. Results of flow cytometry were analyzed with Flowing Software 2.5.1. Statistical analysis was performed with GraphPad Prism 5 or STATISTICA version 7.1 (StatSoft, Inc., Tulsa, OK, USA). Uniform significance level was used through the entire text: * *p* < 0.05.

### 3.2. Drugs

Anthrapyridazones BS-121, C-123 were provided by BS-154 sp. z o.o. (Gdansk, Poland). Doxorubicin was kindly provided by The Institute of Biotechnology and Antibiotics (Warsaw, Poland), whereas mitoxantrone was obtained from Pharmaceutical Research Institute (Warsaw, Poland). Following compounds were purchased from Sigma-Aldrich (Louis, MO, USA): 7-aminoactinomycin (A9400), idarubicin (I1656), etoposide (E1383), C-1305, ethanol, DMSO. All drugs were dissolved in DMSO to concentrations of 2 to 10mM and stored at −20 °C. 7-AAD was dissolved in MeOH:H_2_O (4:6) and stored at 4 °C.

### 3.3. Cell Cultures

HL-60 and A-549 were from ATCC. The HL-60 and A-549 cell lines were cultured in RPMI-1640 medium and HEK-293 Eagle’s Minimum Essential Medium. All cell lines were supplemented with 10% FBS (Sigma-Aldrich F7524), 2 mM L-glutamine (Sigma-Aldrich G8540), and the antibiotics penicillin (100U/mL) and streptomycin (42.4 mg/mL) at 37 °C in a humidified atmosphere of 5% CO_2_ and 95% air. All cell lines were routinely screened for mycoplasma contamination.

### 3.4. Transmitted Light Microscopy

Cells were washed once with PBS, resuspended in PBS, and cytospun on a glass slide (850 RPM, 4 min) using CytoFuge 2 (StatSpin). Cells were fixed with 70% ethanol and stained with May-Grünwald-Giemsa stain.

### 3.5. Clonogenicity

The medium MethoCult Classic (H4434; Stem Cell, Vancouver, BC, Canada) was portioned into tubes ranging from 4 mL to 15 mL Falcon tubes. In each tube, 400 µL of cell suspension at a density of 15 × 10^3^ cells per mL of HL-60 sublines in non-supplemented RPMI 1640 medium (Corning, Corning, NY, USA) was added. Samples were intensively vortexed for 2–4 s and left for 10 min to allow the removal of air bubbles from the growth medium. Then, using a syringe with a capacity of 5 mL Luer-lock (KD Medical, Berlin, Germany) and a blunt 18G needle (BD Falcon, Corning, NJ, USA), 1.1 mL of medium was added to the wells of a 6-well plate (Corning, Corning, NY, USA). The plates were closed in microchambers, ensuring a humid atmosphere. After 1 week of incubation under standard culture conditions, transmitted light scans of the wells were made using a IX83 microscope (Olympus, Center Valley, PA, USA). The micrographs were analyzed in the Fiji program.

### 3.6. Drug Sensitivity Assay

Cells were seeded in 48-well plates in 1mL of culture medium and treated for 120 h with compounds or vehicle control. Next, MTT was added to the final concentration of 0.4 mg/mL, and plates were incubated for 4 h at 37 °C. The medium was aspirated, and precipitated formazan crystals were dissolved in 1 mL of DMSO. Absorbance was measured at l = 540 nm with the Asys UVM340 microplate reader.

### 3.7. Western Blotting

Western blotting cells were lysed in RIPA buffer (5 mM EDTA, 0.1% (*w*/*v*) SDS, 1% (*v*/*v*) NP-40, 0.5% (*w*/*v*) sodium deoxycholate, 150 mM NaCl, 50 mM Tris pH 7.4), with the addition of the commercially available protease inhibitor cocktail (Roche, Basel, Switzerland) and phosphatase inhibitors: 50 mM NaF, 50 mM β-glycerophosphate, and 1 mM Na_3_VO_4_ (30 min on ice). Lysates were cleared at 16,100× *g* (4 °C, 10 min) and protein concentration was measured with BCA assay (Thermo Scientific 23227). Equal amounts of protein were prepared in Laemmli Buffer and loaded on polyacrylamide (7.5, 10 or 12%) gels. SDS PAGE gels were resolved in Running Buffer (15 min at constant 100 V and 50 min at 200 V). Proteins were transferred on the pre-wet polyvinylidene fluoride (PVDF) membranes in Transfer Buffer (3 h at constant current of 250 mA, 4 °C). PVDF membranes were blocked in 5% BSA in TBST, and incubated (overnight, 4 °C) with appropriate primary antibodies: Anti-Actin (1:600, Santa Cruz sc-1616), topoisomerase II α, topoisomerase II β. Next, membranes were incubated with secondary anti-mouse, anti-rabbit, and anti-goat antibodies (all at 1:10,000, Jackson ImmunoResearch 715-035-150, 711-035-152, 705- 036-147). X-ray films (Agfa) were developed using enhanced chemiluminescence (SuperWest Pico, Thermo Scientific).

### 3.8. Topoisomerase Inhibition Assays

The topoisomerase IIα (Topogen TG2013) reaction mixture contained 200 ng kDNA, 50 mM Tris-HCl (pH 7.5), 150 mM NaCl, 10 mM MgCl_2_, 5 mM ATP, 0.5 mM DTT, and 30 μg/mL BSA. Reactions were terminated by adding 1% sarkosyl, 5% glycerol, and 0.05% bromophenol blue in ddH_2_O (final concentrations). Cleavage was performed with 200 ng pBR322 in the same buffer and the reactions were terminated by adding 0.3 mg/mL proteinase K in 0.35% SDS, 15 mM EDTA, and topoisomerase digestion (90 min, 50 °C), before adding loading buffer (0.1% SDS, 5% sucrose, 2.5 mM EDTA 0.05% bromophenol blue in ddH_2_O, final concentrations). Samples were resolved in 1% agarose gel in TBE for 12 h at constant voltage of 1 V/cm and current not exceeding 20 mA. Gel was stained with ethidium bromide, destined in TBE, and photographed under UV illumination.

### 3.9. Proteasome Inhibition

The lysis buffer contained 50 mM HEPES-NaOH (pH 7.8), 10 mM NaCl, 1.5 mM MgCl_2_, 1 mM EGTA, 1 mM EDTA, and 250 mM sucrose. The reaction buffer contained 100 µM Suc-LLVY-AMC, 5 mM DTT, 2 mM ATP, and the test compound in 0.5% DMSO.

A-549 cell pellet was suspended in lysis buffer (10 × 10^3^ cells per µL) and then sonicated for 3 s at 40% of maximum amplitude (Branson, Shanghai, China). Lysates were centrifuged for 15 min at 4 °C with at 16.100 g. The supernatant was transferred to fresh tubes. Then, 100 µL of reaction buffer (containing the test compound) and 25 µL of cell lysate were added to the wells of a white 96-well plate. Fluorescence kinetics from released 7-amino-4-methyl coumarin (AMC) fluorochrome (excitation: 360/20 nm, emission: 460/20 nm) was measured on a Spark M10 plate reader (Tecan, Männedorf, Switzerland). The results were analyzed in Excel 365 (Microsoft, King County, WA, USA).

### 3.10. Molecular Computational Simulations

The atomistic model of C-123 was parametrized using the Generalized Amber Force Field (GAFF) [78], with bond and angle parameters refined using the modified Seminario method [79] based on a B3LYP calculation of the Hessian matrix in Gaussian 16. A DNA duplex 14-mer with a 5′-CACTACCTCTGTCG-3′ sequence was generated using X3DNA [80]. The dodecahedron box with a side length of 8.1 nm contained the DNA molecule, 10 copies of C-123, 11,741 TIP3P water molecules, and Na^+^ and Cl^-^ ions ensuring charge neutrality at a physiological concentration of 0.15 M. A single long equilibrium 2.75 μs simulation was performed using Gromacs 2019 [81]. The amber99sb forcefield with the bsc1 correction for nucleic acids [82] was used. The CSVR thermostat kept the temperature at 300 K [83], with the Berendsen barostat maintaining the pressure at 1 bar [84]. Long-range electrostatics were treated using the Particle Mesh Ewald summation, and LINCS allowed the use of a standard 2-fs timestep to integrate the equations of motion. Plumed 2.5 [85] was used to prevent aggregation of individual C-123 molecules, keeping the minimal distance between any pair of central ligand atoms above 0.65 nm and the terminal DNA bases WC-paired. With this setup, multiple modes of interaction between the ligand and DNA could be simultaneously assessed in a qualitative manner even at unphysically high ligand concentrations. Movies S1 and S2 were generated using Molywood [86].

## 4. Conclusions

Research of CSCs based on the etiology of AML has the potential to translate into more effective therapies aimed at eliminating the cell stage responsible for the initiation and relapse of the disease [87]. The hope for achieving a therapeutic breakthrough in AML is associated with a new generation of drugs that selectively target the self-replenishing, most primitive stages of the cancer. It is key that the new chemotherapeutics overcome the resistance induced by ABC transporters and target the proteins responsible for PC survival. A screening platform based on cell density-dependent stage profiles is a useful tool to improve the process of preclinical selection of drugs active against PCs, including LSC HSC-like stages.

As a proof of concept, we showed that cell density-dependent stage profile alterations may also be used to construct screening platforms based on epithelial cancer and non-cancer cell lines. The platform we previously developed enabled the calculation of the PC resistance index of a drug [33]. In this work, we presented an additional problem of dosing resulting from stage differentiation of the cell culture population profile (Figure 2 and Figure 3 and Table 1 and Table 2). The cellular and molecular study of HL-60, A-549, and HEK-293 demonstrates that obtaining robust pharmacological data requires more than the maintenance of identical passaging and cell density conditions throughout the experiment (Figure 1 and Figure 5 and Table 4, Table 5 and Table 6). Although the observed number of distinct developmental stages was lower in A-549 and HEK-293 than in HL-60, the need for strict cell density control is still relevant in non-AML lines and cannot simply be ignored. Importantly, when confronted with a lack of sufficient information about the newly tested substance, it is necessary to keep both cell density and drug concentration constant at the initial stage of the research. By determining the culture stage profile and using chemometric methods for the determination of EC_50_ per cell and *EC_50_ per primitive cell, we minimized the noise due to stage transformations of the cells and were able to support a recommendation to use primitive-rich sublines cultured at low densities for maximum reproducibility (Figure 5 and Table 5). The trends observed in EC_50_ and *EC_50_ calculations are highly informative of stage-specific resistance and can inform mechanistic investigations into the detailed molecular mechanism of action. We also observed that the use of derived EC_50_ values provides a more robust and reliable approach to the calculation of selectivity indices, here calculated against a Primitive HEK-293 subline as a reference healthy cell type (Table 4, Table 5 and Table 6). Finally, we showed how the developed stage profiles can help expand the understanding of a molecular mechanism of action using the example of C-123, a promising drug candidate characterized by strong selectivity against AML cells and activity against their primitive sublines (Figure 5, Table 6, and Supplemental Information/Data).

## Figures and Tables

**Table 1:** Stage profiles of cultured HL-60 sublines. The table presents the cell density-dependent cytological stage profiles of HL-60 based on immunophenotype characteristics and cytological measures (flow cytometry scattering and clonogenity) presented in Figure 2 and Supplementary Figure S1A and defined by Lica et al. [33]. * Primitive. ˚ Maturation.

Cellular Stage	HL-60 Percent of Cell Stage		
	Primitive	Standard	Mature
* LSC-like, * Progenitor-like and * CFU-like	20–25	5–10	1–1.5
* ˚ Blast-like	45–60	25–30	2–2.5
˚ Promyelocyte-like	5–30	10–15	5–7
˚ Myelocyte-like	2.5–5	40–50	10–20
Senescent	2.5–5	5–10	72–80

**Table 2:** Proposed cell density-dependent A-549 cytological stage profile. Similar to the AML and hematopoietic cells, A-549 cells also show a degree of variability characterized cytology by phenotype- and likely stage-dependent ability to metastasize in mice xenograft models [38]. Based on analysis of the determined cytological parameters such as cell size, nucleus size and morphology, pH of cytoplasm, FSC/SSC, phosphatidylserine presentation, DNA distribution, generation of ROS (Figure 3A,B), as well as stage-specific characteristics of lung malignancies [39,40,41,42,43], Table 2 compiles fractional populations of A-549 stage forms derived at different culture cell densities. The demonstrated presence of cancer stem cells in A-549 culture [44,45,46,47,48], increase of this fraction depending on the line passaging [49], and ability to induce epithelial-mesenchymal transformation suggest the presence of progenitors and/or CFU-like stages. Similar to the presented cytological characteristics of carcinomas, blast-like stages can also be observed in A-549 [50,51,52,53,54]. The characteristics of ROS-positive aging cells in Mature A-549 are analogous to those described in the literature [55,56,57,58]. * Primitive. ˚ Maturation.

Cellular Stage	A-549 Percent of Cell Stage		
	Primitive	Standard	Mature
* CSC-like and * Progenitor-like	70–90	30–70	20–35
*˚ Blast-like	10–25	30–60	10–15
Senescent ROS negative	0.5–5	5–2	25–35
Senescent ROS positive	0–0.5	5–8	25–35

**Table 3:** Molecular targets of tested drugs and their activity toward primitive cells (PCs). Drugs used in this study were classified according to their strength of interaction (weak/medium/strong), main mechanisms of action, and activity toward PCs (*EC_50_ = EC_50_ relative normalized to PC = high: <1.5, medium: >1.5 <10, low > 10) according to literature and this study (also see Supplementary Text ST1, ST3 and ST4). Among drugs with known mechanisms of action, C-123 and bortezomib display high activity toward PCs. Both compounds uniquely inhibit proteasomal degradation, suggesting that this process is critical for PCs’ survival. Unsatisfactory PC specificity of idarubicin, doxorubicin, and C-1305 is likely a result of primitive cells lacking 1 of the targets (low topoisomerase IIα expression) and their high reduction potential provided by their high intracellular glutathione content (as reported by the authors of [33]), protecting cells from ROS damage.

Compound	Main Targets		
	Strength of Interaction	Mechanism of Action	PC Activity
C-123	Strong	DNA Breaks DNA Intercalation Proteasome Inhibition	High
Bortezomib	Strong	Proteasome Inhibition	High
Idarubicin	Strong	Reactive Oxygen Species Topoisomerase IIα Inhibition DNA Breaks DNA Intercalation	Medium
Doxorubicin	Strong	Topoisomerase IIα Inhibition DNA Breaks DNA Intercalation	Medium
C-1305	Strong	Topoisomerase IIα Inhibition DNA Intercalation	Medium
DMSO	Medium	Broad, Not fully determined	Medium
Ethanol	Weak	Broad, Not fully determined	Medium

**Table 4:** Inhibitory and effective concentrations calculated for HL-60. (**A**) IC_50_ at different initial densities for 3 HL-60 sublines. (**B**) IC_50_ averages. IC_50_ average calculated for total cell number. *IC_50_ average calculated for primitive cell number. Drug activity units corresponding to (**A**). (**C**) EC_50_ at different initial densities for 3 HL-60 sublines. (**D**) EC_50_ averages. EC_50_ average calculated on total cell number. *EC_50_ average calculated on primitive cell number.

**A**	**Inhibitory Concentration IC_50_**											
**Culture Density** **[10^3^ Cells per mL]**	**C-123 [nM]**			**Idarubicin [nM]**			**DMSO [Percent]**			**Ethanol [Percent]**		
	**Primitive**	**Standard**	**Mature**	**Primitive**	**Standard**	**Mature**	**Primitive**	**Standard**	**Mature**	**Primitive**	**Standard**	**Mature**
5	19.0 ±0.4	18.2 ±0.3	18.2 ±0.2	6.8 ±0.8	2.0 ±0.1	0.7 ±0.1	2.6 ±0.3	1.3 ±0.2	0.5 ±0.1	2.4 ±0.2	1.7 ±0.2	1.0 ±0.1
25	56.3 ±0.2	57.3 ±0.7	55.4 ±0.5	12.4 ±2.0	5.1 ±0.7	2.5 ±0.2	4.8 ±0.4	2.6 ±0.2	1.2 ±0.1	4.2 ±0.2	2.1 ±0.1	1.6 ±0.1
100	201.6 ±0.5	198.7 ±1.0	199.1 ±0.8	32.7 ±0.6	16.2 ±2.4	5.8 ±1.5	10.1 ±1.5	4.6 ±0.5	2.1 ±0.3	4.1 ±0.4	1.5 ±0.2	0.4 ±0.1
**B**	**IC_50_ average calculated for total cell number [10^3^ cells per mL]**											
	5	25	100	5	25	100	5	25	100	5	25	100
	18.5	56.3	199.8	3.2	6.7	19.7	1.5	2.9	5.6	1.7	2.6	2.0
	*IC_50_ average calculated for primitive cell number											
	Primitive	Standard	Mature	Primitive	Standard	Mature	Primitive	Standard	Mature	Primitive	Standard	Mature
	92.3	91.4	90.9	18.8	7.8	3.0	5.8	2.8	1.3	3.6	1.8	1.0
**C**	**Effective Concentration EC_50_**											
	**[fMper cell]**			**[fM per cell]**			**[µM per cell]**			**[µM per cell]**		
5	3.8	3.6	3.6	1.4	0.4	0.1	67.7	33.9	12.1	103.5	75.6	44.1
25	2.3	2.3	2.2	0.5	0.2	0.1	24.7	13.5	6.2	36.2	18.6	14.1
100	2.0	2.0	2.0	0.3	0.2	0.1	13.0	5.9	2.7	8.9	4.6	0.8
**D**	**EC_50_ average calculated for total cell number [10^3^ cells per mL]**											
	5	25	100	5	25	100	5	25	100	5	25	100
	3.7	2.3	2.0	0.6	0.3	0.2	37.9	14.8	7.2	74.4	23.0	4.8
	*EC_50_ average calculated for primitive cell number											
	Primitive	Standard	Mature	Primitive	Standard	Mature	Primitive	Standard	Mature	Primitive	Standard	Mature
	2.6	2.6	2.7	0.1	0.3	0.7	7.0	17.8	35.1	19.7	32.9	49.5

**Table 5:** Stage profiles of A-549 affecting the results of proliferative assays. IC_50_ (**A**) and EC_50_ (**B**) as well selectivity indices of compounds active against CSCs (C-123, bortezomib) and topoisomerase poisons (C-1305, doxorubicin) [67,68,69,70,71]. Initially, 0.25 × 10^3^ (A-549) or 0.5 × 10^3^ (HEK-293) cells were seeded in a single well of the 96-well plate. Due to a likely lack of anti-CSC activity (2-fold differences in IC_50_ between Standard and Mature sublines for C-1305 and doxorubicin), the MTT assay was not performed with the Primitive subline.

**A**	**IC_50_ [nM]**						
**Compound**	**A-549**			**HEK-293**	**Selectivity Index**		
	Primitive	Standard	Mature	Primitive	Primitive	Standard	Mature
C-123	45.6 ± 1.8	39.8 ± 2.0	41.3 ± 1.3	27.6 ± 4.5	0.6	0.7	0.7
Bortezomib	13.5 ± 0.7	14.3 ± 0.9	15.3 ± 0.9	2.2 ± 0.1	0.2	0.1	0.1
C-1305	N.d.	18.5 ± 1.4	33.3 ± 2.6	18.5 ± 2.8	N.d.	1	0.6
Doxorubicin	N.d.	39.7 ± 3.3	61.8 ± 3.8	51.7 ± 9.0	N.d.	1.3	0.8
**B**	**EC50 [fM per cell]**						
	**A-549**			**HEK-293**	**Selectivity Index**		
	Primitive	Standard	Mature	Primitive	Primitive	Standard	Mature
C-123	36 ± 1	32 ± 2	33 ± 1	11 ± 2	0.3	0.3	0.3
Bortezomib	11 ± 1	11 ± 1	12 ± 1	1 ± 0	0.1	0.1	0.1
C-1305	N.d.	15 ± 1	27 ± 2	7 ± 1	N.d.	0.5	0.3
Doxorubicin	N.d.	32 ± 3	49 ± 3	21 ± 4	N.d.	0.7	0.4

**Table 6:** Selectivity indices of bortezomib and C-123 against leukemic HL-60 cells using HEK-293 as reference. (**A**) *IC_50_, *EC_50_ and selectivity index calculated for bortezomib. IC_50_ and EC_50_ values for bortezomib measured for HEK-293 are reported in Table 5. (**B**) Selectivity indices calculated using IC_50_ for C-123. (**C**) Selectivity indices calculated using EC_50_ for C-123. IC_50_ and EC_50_ values for C-123 measured using HL-60 are reported in Table 4.

**A**	**Bortezomib Selectivity Index**			
**Culture Density** **[10^3^ Cells per mL]**	**Primitive HL-60** *IC_50_	**SI_IC50_**	**Primitive HL-60** *EC_50_	**SI_EC50_**
0.5	14.75 ± 1.02	0.15	2.95 ± 0.20	0.34
**B**	**C-123 Selectivity Index calculate per IC_50_**			
	**Primitive HL-60**	**Standard HL-60**		**Mature HL-60**
5	1.5	1.5		1.5
25	0.5	0.5		0.5
100	0.1	0.1		0.1
**C**	**C-123 Selectivity Index calculate per EC_50_**			
5	14.6	15.2		15.2
25	24.5	24.1		25.0
100	27.4	27.8		27.8

## Data Availability

Data available in a publicly accessible repository.

## Supplementary Materials

The following are available online at https://www.mdpi.com/article/10.3390/ijms22094931/s1: Supplementary Text ST1, ST2, ST3 and ST4; Supplementary Figure S1, S2, S3 and S4; Supplementary Table S1; Supplementary Video-Movie SVM1, SVM2, SVM3 and SVM4; Supplementary Methodology (Microscopy Equipment, Transmitted Fluorescence Microscopy, Time Laps Microscopy, Comet Assay, *β*-Galactosidase Assay, Immunofluorescence, Cytometry); Supplementary References SR1-22.

## Abbreviations

ABC	ATP-Binding Cassette
AML	Acute Myeloid Leukemia
A-549	lung adenocarcinoma cell line
CFU-like	Colony Forming Unit-like
DMSO	dimethyl sulfoxide
EC	Effective Concentration
FSC	laser light forward scattering
HSC	Hematopoietic Stem Cell
HEK-293	human embryonic kidney cells
HL-60	human myeloid leukemia cells
IC	Inhibitory Concentration
LB	left bottom
LIC	Leukemia Initiation Cell
LT	left top
LSC HSC-like	Leukemic Stem Cell HSC-like
MDR	Multi-Drug Resistance
MGG	May-Grunwald-Giemsa
OPP-like	Oligopotent Progenitor-like
PC	primitive cell
RB	right bottom
ROS	Reactive Oxygen Spices
RT	right top
SI	Selectivity Index
SSC	laser light side scattering
