# Supplementary material for: Effective Drug Concentration and Selectivity Depends on Fraction of Primitive Cells
Source: Int J Mol Sci. 2021 May 6;22(9):4931. doi: 10.3390/ijms22094931 (PMC8125035; doi:10.3390/ijms22094931)
Supplement: Supplementary file 1 [file ijms-22-04931-s001.zip › supplementary/Supplementary -final.pdf]

Supplementary information

Supplementrary Text ST1.

Anthrapirydazones

Anthrapirydazones are rationally designed analogs of anthraquinones that demonstrated low affinity toward ATP Binding Cassette (ABC) transporters in MDR1- and MRP1-resistant HL-60 lines, as well as cytotoxicity in the nanomolar range [SR1]. Both anthraquinones and anthrapyridazones interact with NADPH oxidoreductases, which disturb cell redox potential [SR2]. The interaction of anthrapyridazones with the environment generates a highly reactive semiquinone radical, thus increasing the production of reactive oxygen species (ROS), in particular in lysosomes. However, more promising outcomes could be achieved by preserving nuclear cytotoxicity while reducing ROS production, as recognized in clinically applied cytostatic anthracyclines that harbor minimal structural modifications modulating ROS generation. Four of the anthrapyridazones previously tested by us, PDZ-7, PDZ-14, C-123, and C-155, did not generate ROS but remained cytotoxic [Misiak *et al*. 2017, Lica *et al*. 2018]. Out of these, C-123 and C-155 were active against HL-60 primitive stages, which include LSC HSC-like. The activity of C-123 and C-155 toward primitive stages was shown for HL-60 sublines using MTT test [Lica et all. 2018]. For C-123, it was further confirmed by cell cycle monitoring and microscopic observations. Interestingly, C-123 but not C-155 exhibited low toxicity (MTD >100mg/kg^{-1}) and did not affect the morphology of blood cells in mice [Lica et all. 2018]. Thanks to desirable properties such as very low ROS generation and little interference with mitochondrial function, warranting low overall toxicity, C-123 is a promising novel agent, nonspecifically active against primitive stages.

Supplementary Text ST2.

Detailed cytological characterization of HL-60 sublines

Immunophenotyping and Morphology. Our microphotographs show that the number of cells characterized by small size, highly condensed chromatin and highly basic cytoplasm is consistent with the stage-specific immunophenotype (CD34$^{very\text{-}high}$, CD117$^{very\text{-}high}$, HLA-DR^{high}, CD123^{high}, CD38$^{very\text{-}low}$, CD45$^{very\text{-}low}$), as well as with light scattering profiles: side SSC and forward FSC (Figure Supplementary Figure S1A) [SR3–6]. In the SSC/FSC dot-plot, the LSC HSC-like population is located in the area with low values of SSC and FSC (Supplementary Figure S1A). We speculate that the considerable width of the population distribution along the SSC axis results from the cellular divisions of the LSCs HSC-like [SR7–13]. The division of Oligopotent Progenitor-like (OPP-like) cells (formed by asymmetric LSC HSC-like division) results in the formation of CFU-like cells with different SSC/FSC characteristics, further accelerating proliferation and differentiation of the colony (Figure 2A and Supplementary Figure S1A). The formation of blast-like cells from by CFU-like cells initiates colony maturation (Figure 2A). The resulting blast-like maturing promyelocyte-like stages do not differentiate but rather divide symmetrically in the formation of two myelocyte-like cells (Figure 1C).

ABC Transporters – Hoechst 33342 Cellular Export. PCs in AML are characterized by increased expression of ABC transporters, which protect the cell against the influence of xenobiotics [Borowski *et al.* 2005]. The efficiency of export of cytotoxic substances by ABC transporters can be measured using hoechst 33342 [SR12-13]. It was already reported in the literature that one of the characteristic of primitive cells is an increased level of ABC transporters. Correspondingly, AML cell differentiation is associated with a decrease in the expression level of ABC transporters. We want, however, to emphasize that the issue of cell stemness is much more complex. Anthrapyridazones were designed to be bad substrates of ABC transporters, as confirmed in cell lines overexpressing specific ABC transporters [SR1]. Despite that, primitive stages of HL-60 still show resistance to most anthrapyridazones [Lica *et al.* 2018]. Here we present an approach allowing for consistent drug candidate evaluation, taking into account alterations in cytological stage profiles that occur in cell cultures.

The vital staining fluorescence micrograph presented in Supplementary Figure S1B shows HL-60 stained with JC-1 and hoechst 33342. Small and extensively proliferating (high energy demand –

high mitochondria signal) cells remove hoechst 33342 from their interior more effectively than larger ones at slower-proliferating stages (Supplementary Figure S1B and S2A). In accordance with the hierarchical morphological classification presented in Figure 1 A-B, Primitive HL-60 cells retained higher expression of ABC transporters.

Colony Forming Assay. The results of the colony-forming assay shows that the Primitive HL-60 cells were the most, and Mature HL-60 the least capable of forming colonies (Figure 2B). Clonogenicity in the Primitive subline was approx. 18%, i.e. 2x higher than for Standard and 12x higher than for Mature HL-60 (Figure 2B). The fraction of cells capable of forming colonies in HL-60 sublines (Figure 2B) correlates with the percentage of PCs, especially with OPP/CFU-like stages in primitive HL-60 (Supplementary Figure S1A and Table 1) [Lica *et al.* 2018]. Microphotographs of HL-60 colonies stained with MGG show the extent of developed colony structure differentiation (Figure 2A). The colonies that formed during the clonogenicity assay were located near to cells with an LSC HSC-like morphology (Figure 2A-B). Regardless of the size or maturity of the colony, CFU-like stages were observed in nearly all colonies (occasionally during cell fusion; Figure 2A). Changes in fractions of individual stages during cell proliferation, as well as the demonstrated ability to seed tumor formation in mice, both agree with data obtained in other studies including mathematical modeling, strongly suggesting that LICs originate from the LSC HSC-like in HL-60 [SR14–18].

Topoisomerase II α and β. Topoisomerase poisons based on anthracyclines are commonly used drugs in AML therapy [SR19]. On a cellular level, the sensitivity to anthracyclines correlates with the level of topoisomerase IIα and β [SR19-20]. As shown in Figure 2C, Primitive HL-60 has almost 2.5 times lower levels of topoisomerase IIα level but approx. 5 times higher levels of topoisomerase IIβ than Standard HL-60. This agrees with previous reports showing that PCs in AML are characterized by a higher level of topoisomerase II β and lower of topoisomerase IIα.

Supplementrary Text ST3.

The cellular effect of C-123, idarubicin, DMSO and ethanol on HL-60

In our previous work, a wide panel of drugs with various mechanisms of action was evaluated on HL-60 cells [Lica *et al.* 2018]. By comparing the LSC-enriched with a parental cell line, the lowest resistance indices were obtained for proteasome inhibitors – bortezomib (already shown to be active against both LSCs and CSCs) and MG-132 – and a novel group of anthrapyridazones, C-123 and C-155. C-155 and MG-132 were excluded from further investigations as they proved either generally toxic in mice (C-155) or showed neurotoxicity (MG-132). We hence focused on C-123, reporting the DNA distribution, BrdU incorporation, dsDNA break induction as well as phosphatidylserine presentation assays performed in HL-60; the results were compared to classic anthracyclines, doxorubicin and idarubicin [Lica *et al.* 2018]. The direct interaction of C-123 with DNA did not result in a G_2/M cell cycle block observed for other DNA-damaging compounds. The lack of significant difference in drug sensitivity of topoisomerase II knockout cell lines compared with the wild-type one suggests that the cytotoxic effect is independent of topoisomerase II function [Lica *et al.* 2018]. However, we observed that a fraction of cells accumulated ROS-independent DNA breaks triggered cell death.

In this report, we use C-123, idarubicin, doxorubicin, DMSO and ethanol as reference compounds. Idarubicin and doxorubicin are approved drugs, still widely used in treatment of AML and non-small cell lung cancer. They also share the major mechanism of action, being topoisomerase II poisons. However, they differ in that idarubicin has a more potent ROS generation activity, as demonstrated in HL-60 [Lica *et al.* 2018]. In turn, ethanol and DMSO act as co-solvents, affecting the environment rather than individual molecular targets, and hence require concentrations orders of magnitude higher to exert a biological effect [SR21].

As a result of idarubicin-induced topoisomerase inhibition and ROS generation, a significant fraction of surviving HL-60 cells are at polyploid and multilobular stages (Supplementary Figure S2). Cytologically, this fraction resembles stages of megakaryocyte-like development and other cell types, especially erythroblast-like (Supplementary Figure 2A). DMSO induces differentiation of AML cells, leading to loss of proliferative capacity and death of senescent cells. Ethanol accelerates the process of cell differentiation but is unable to induce it on its own. We observe that the addition of 2.5 and

5% DMSO to HL-60 mainly causes death of cells at aging stages, and induces differentiation of remaining cells into many subtypes, predominantly megakaryocytic-like fractions (Supplementary Figure S2). This effect of cell death induction and enhanced differentiation was also observed upon ethanol addition.

Supplementrary Text ST4.

Cellular effect and mechanistic studies of C-123 on A-549

Interactions with DNA. Previous studies showed that the cytotoxic effect caused by C-123 in HL-60 and A-549 is independent of ROS [Misiak *et al*. 2017, Lica *et al*. 2018]. Despite this, after 24 hours of exposure to C-123, about 60% of cells becomes positive for double strand DNA breaks in HL-60, while only <2,5% in A-549 (Supplementary Figure S4A), as indicated by increased phosphorylation of histone H2AX and activation of DNA damage response. To study the mechanism of DNA damage induction by C-123, we compared its action with etoposide (VP-16), a topoisomerase II inhibitor used in cancer treatment that quickly induces high amounts of double stranded DNA breaks independent of ROS [SR14]. Our analysis of DNA distribution and BrdU incorporation performed on A-549 cells treated with C-123 show significant differences compared to etoposide after 24 and lower difference at 48 hours (Supplementary Figure S4A). In comparison to antrapyridazone BS-121, a compound that generates high levels of ROS in HL-60 and low in A-549 cells, C-123 generated ROS at low level in both HL-60 and A-549 [Misiak *et al*. 2017, Lica *et al*. 2018]. Nevertheless, the comet assay showed much more abundant DNA breaks in A-549 cells treated with C-123 than with BS-121 (Supplementary Figure S3), confirming that ROS are not responsible for the cytotoxicity of C-123.

Cellular Senescence and Cytoskeleton. Using the β-galactosidase senescence assay with light micrographs for A-549 cells (Supplementary Figure S4B) as well as time lapse microscopy, we showed that C-123 is capable of inducing senescence within 24 hours, like BS-121 and doxorubicin. Senescence induction was confirmed with white light time-lapse microscopy at a concentration corresponding to IC_{90} (Supplementary Video Movie SVM3). The preferential induction of senescence instead of apoptosis is typical for A-549. C-1305 was used as a control (Supplementary Video Movie SVM4). Since a related compound from the antrapyridazone group, PDZ-7, has been shown to predominantly act through inhibition on actin polymerization [Misiak *et al*. 2017), we also tested for possible similarities in the mechanisms of action. To this end, we performed immunofluorescence microimaging of β-tubulin and actin after 24 hours of incubating A-549 cells with C-123, but found no significant morphological disturbances in these structures (Supplementary Figure S4C).

Supplementary Table

Supplementary Table S1. Basal C-123 molecular mechanism of action – Summary Table. LEGEND, *External Membrane:* Integrity (white light and fluorescence microscopy observations) – low effect (lack of quickly induced loss cell integrity), Phosphatidylserine Presentation (level of annexin V positive cells during 24 hours measured by flow cytometry), + more than 15% lower than 30%, ++ more than 30% lower than 60%, +++ more than 60%; *Lysosomes:* Structural Damage (white light and fluorescence microscopy observations as well as flow cytometry analysis) - low effect (lack of significant shape deformations), ROS Generation (level of ROS positive cells measured during 24 hours by flow cytometry) + more than 15% lower than 30%, ++ more than 30% lower than 60%, +++ more than 60%; *Cytoskeleton:* Structural disruption of α-Tubulin and β-Actin (immunofluorescence microscopy observations) – low effect (lack of significant damage); *Senescence and Apoptosis*: Senescence – for HL-60 measured using clinically used cytostatic such as idarubicin/doxorubicin/daunorubicin as reference +++, for A-549 measured based on counts of β-gal positive cells compared to doxorubicin and BS-121 +++; Apoptosis – for HL-60 measured using clinically used cytostatics such as idarubicin/doxorubicin/daunorubicin as reference +++, for A-549 the required concentrations are not achievable in the therapeutic regime [Sabisz *et al*. 2009]; *Nucleus and DNA:* Nuclear Morphology (white light and fluorescence microscopy observations) – low effect (lack of rapidly induce schappe deformations), DNA Distribution and Synthesis (changes of cells level in cell cycle phase measured during 24 hours by flow cytometry by propidium iodide stain and BrdU incorporation) – low effect (lower than 10% change of value). Generation of double-strand DNA

breaks - DSDB: (level of γHA2X positive cells during 24 hours measured by flow cytometry) + more than 15% lower than 30%, ++ more than 30% lower than 60%, +++ more than 60%, *Enzymes/Molecular Targets* (*in vitro*): Topoisomerase II Inhibition (inhibition compared to strong concentration dependent topoisomerase poison BS-121 +++, PDZ-7 +++); Proteasome Inhibition (activity percent of strong inhibitor – bortezomib) more than 10% lower than 30%,++ more than 30% lower than 60%, +++ more than 60%; *Molecular Modeling*: – (not observed), + (observed), ++ (strong tendency).

C-123 Molecular Mechanism of Action

**A**	**Cellular Study**	
**Organelle/Structure**	**Cell Line**	
	**HL-60**	**A-549**
External Membrane		
Integrity	-	-
Phosphatidylserine Presentation	+++	+++
Lysosomes		
Structural Damage	-	-
ROS Generation	-	-
Cytoskeleton		
αTubulin	Nd	-
βActin	Nd	-
Senescence and Apoptosis		
Senescence	+++	+++
Apoptosis	+++	-
Nucleus and DNA		
Nuclear Morphology	-	-
DNA Distribution and Synthesis	-	-
Generation of DSDB	++	+

**B**	**In Vitro Study and Molecular Computational Modeling**
**Technique *In Vitro***	**Effect**
Enzymes/Molecular Targets	
Topoisomerase II Inhibition	++
Proteasome Inhibition	+
Molecular Computational Modeling	
Minor Grove Binding	++
Major Grove Binding	+
Intercalation	+

Supplementary Figures

Supplementary Figure S1.

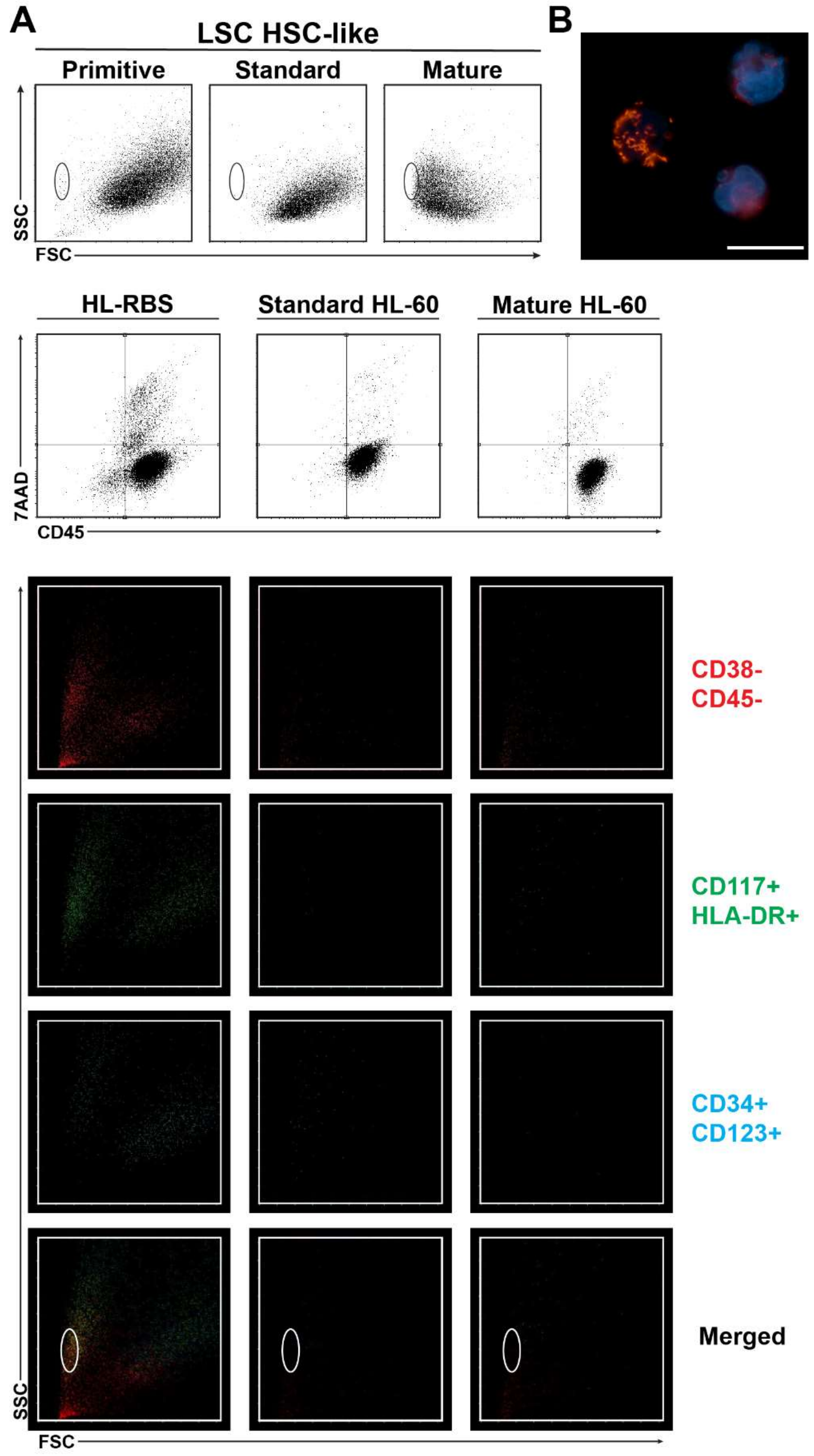

Supplementary Figure S1. (**A**) Gating for HL-60 primitive cells. $CD34^{very\text{-}high}$, $CD117^{very\text{-}high}$, $HLA\text{-}DR^{high}$, $CD123^{high}$, $CD38^{very\text{-}low}$, $CD45^{very\text{-}low}$. (**B**) Export of hoechst 33342 from HL-60 cells. The fluorescence micrograph of living cells shows the accumulation of hoechst 33342 (blue). The intensely proliferating cells (high mitochondrial signal - JC1 (orange)) of small size compared to the larger and slower proliferating cells remove hoechst 33342 more efficiently.

Supplementary Figure S2.

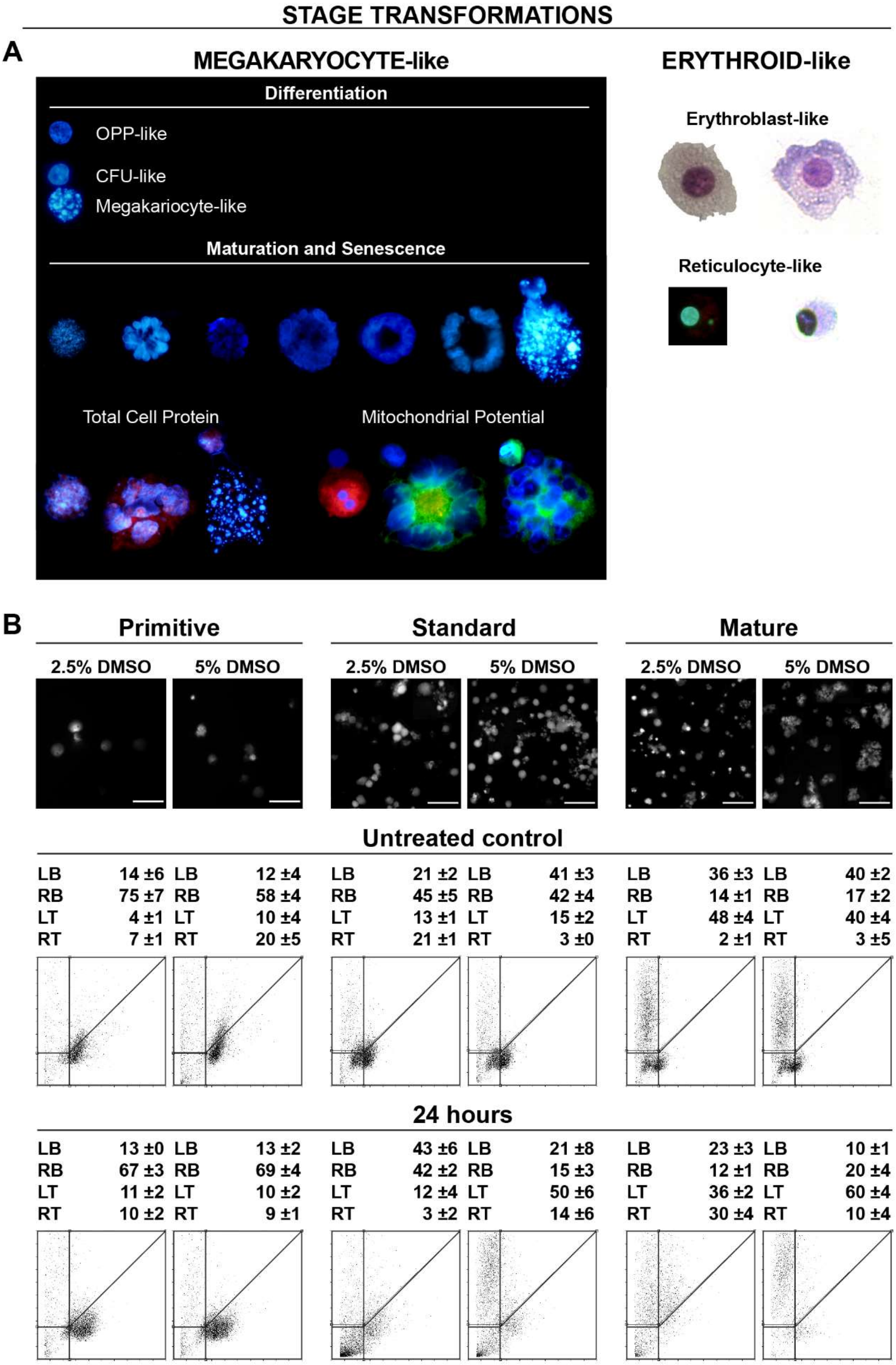

Supplementary Figure S2. Stage forms of HL-60. (**A**) Micrographs of HL-60 showing idarubicin-, ethanol- and DMSO-induced HL-60 differentiation into megakaryocyte and erythroblast-like cells. Nuclei are shown in blue (hoechst 33342), total protein in red (sulforhodamine B) [SR22], mitochondrial potential in orange/green (JC-1). (**B**) HL-60 laser cytometric scattering following DMSO treatment. The values are averages ± SD of n = 3 independent experiments. LB – left bottom, RB – right bottom, LT – left top, RT – right top.

Supplementary Figure S3.

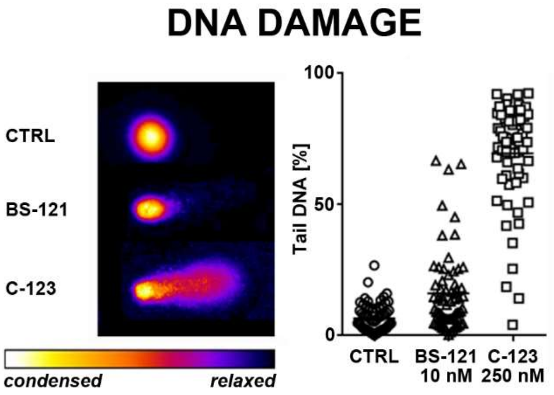

Supplementary Figure S3. DNA damage – A-549 Comet Assay. Induction of DNA breaks by anthrapyridazones BS-121 and C-123. The compounds were added at IC_{90}. The colormap indicates the relative intensity of the fluorescence corresponding to chromatin compaction level.

Supplementary Figure S4.

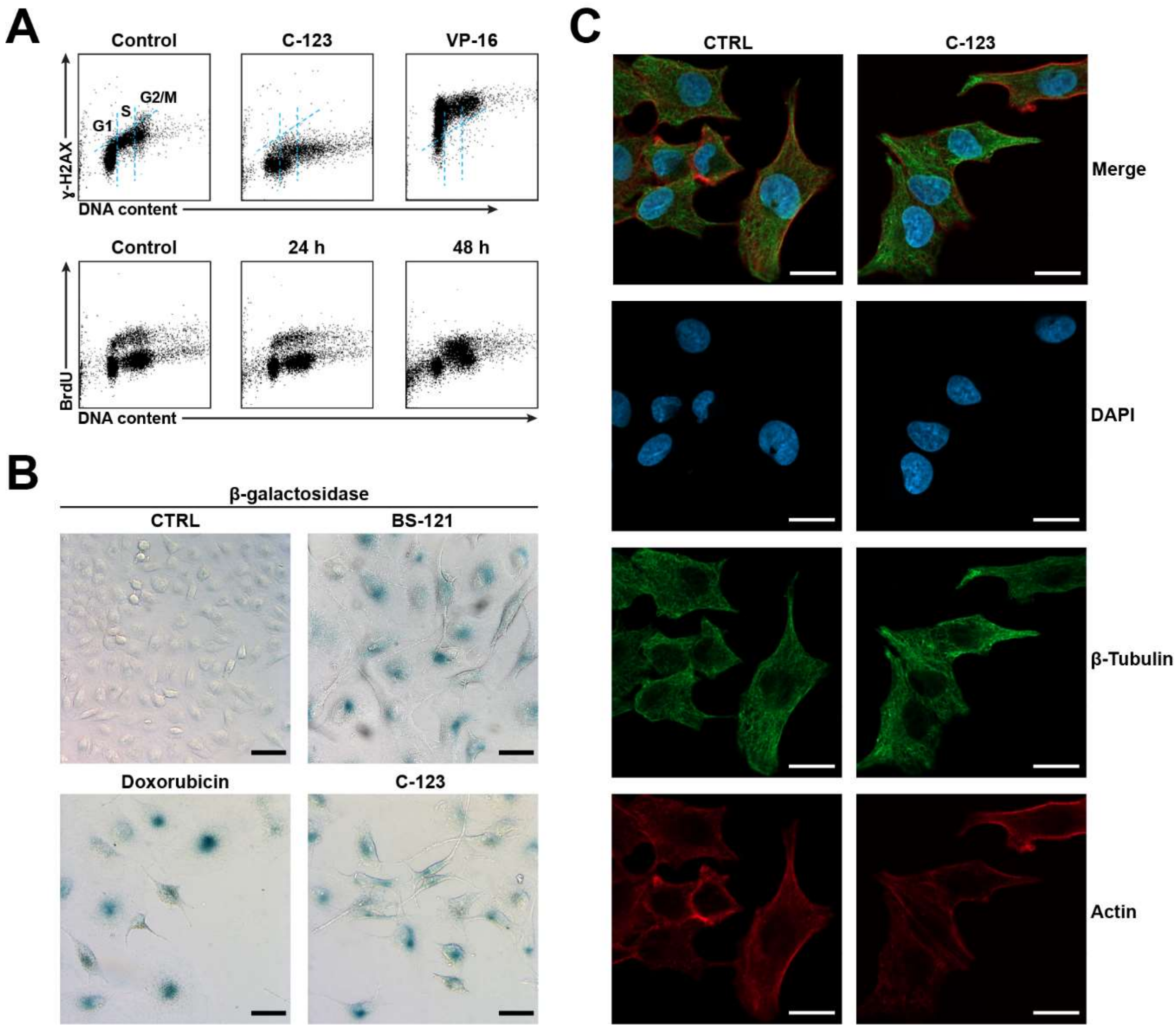

Supplementary Figure S4. Cellular studies of C-123 on A-549. (**A**) Top: generation of double-stranded DNA breaks by C-123. VP16 was used as a positive control. Bottom: disturbance of DNA synthesis by C-123. The compounds were added at IC_{90}. (**B**) Induction of senescence in A-549 cells after 24 hours – β-galactosidase (blue). (**C**) Cytoskeleton and nuclear morphology of A-549 cells treated with C-123 at IC_{90}.

Supplementrary Methodology

Microscopy Equipment

The U-RFL-T mercury burner was used for illumination. The fluorescence microscopy U-MWU filter (Exc. 330–385, Em. 420—) (Olympus, Tokio, Japan) was used for hoechst 33342 (Sigma-Aldrich B2261), the U-MNB filter (Exc. 470–490, Em. 500IF—) (Olympus, Tokio, Japan) was used for acridine orange (Sigma-Aldrich A6014), U-MNIB filter (Exc. 530–550, Em. 590—nm) (Olympus, Tokio, Japan) was used for JC-1 (Thermo Fisher Scientific T3168). The U-MNV filter (Exc. 400–410, Em. 455—nm) (Olympus, Tokio, Japan) sul-forhodamine B (Thermo Fisher Scientific S1307*)* (Exc. 530–550, Em. 590—nm) (Olympus, Tokio, Japan) was also used.

Transmitted Fluorescence Microscopy

The nuclei of live cells were stained with 4 mg/mL hoechst 33342 (Sigma-Aldrich B2261) for 5 minutes acridine orange (Sigma-Aldrich A6014) was used to stain nuclei and lysosomes (1 mg/mL, 10 minutes), whereas mitochondria were stained with JC-1 (Thermo Fisher Scientific T3168) and total level of proteins with sulforhodamine B (Thermo Fisher Scientific S1307). Following a single PBS wash, cells were resuspended in PBS and cytospun on a glass slide (850 RPM, 4 minutes) using CytoFuge 2 (StatSpin).

Time Laps Microscopy

For time-lapse experiment, A-549 cells were seeded into cell-culture treated 96-well plates (at 0.25 x10^3 cells per well) and allowed to attach overnight. The compounds were then added at serial dilutions and the plate was placed into incubation chamber mounted on IX83 motorized inverted microscope (Olympus, Tokyo, Japan). Standard culture conditions were maintained during the experiment. The microphotographs were acquired every 10 minutes using 10x plan-fluorite objective. The data was processed in cellSens Dimension 1.18 (Olympus, Tokyo, Japan).

Immunophenotyping

Immunophenotyping was performed in the Department of Clinical Immunology (Medical University of Gdansk, Poland) according to the EuroFlow standardization of flow cytometer instrument settings. Stadial standards of surface markers were applied according to the DSMZ cell line database (https://www.dsmz.de (24.06.2018)) and lit-erature [Lica *et al*. 2018].

Comet Assay

Cells were treated with indicated drugs for 6 hours, collected and washed once with cold PBS. Cells were then suspended in 0.5 % low melting point agarose, spread on slides (ca. 5 x10^3 cells per 22x22 mm coverslip) and lysed overnight in [1 % triton X-100, 2.5 M NaCl, 100 mM EDTA, 10 mM Tris, pH 10.0] at 4°C. Slides were resolved at 0.75 V/cm for 30 minutes at 4°C and neutralized by washing 3 times with [10 mM Tris-HCl, pH 7.5] for 5 minutes each and once with ddH2O. DNA was fixed with 70 % ethanol for 5 minutes, stained with 1 μg/ml DAPI for 30 minutes and viewed under fluorescent microscope. Comets were scored using AutoComet plugin for ImageJ.

β-Galactosidase Assay

A-549 cells were plated in 35 mm Petri dishes (100 x10^3 cells each). The next day, the medium was removed and a fresh medium containing the drug was added. Drug exposition was continuous and lasted 120 hours. Cells were washed twice with warm PBS, fixed in FG buffer (2% formaldehyde, 0.2% glutaraldehyde in PBS) for 5 minutes at RT. After the next two washes with PBS, cells were stained with 1 mg/mL X-Gal in SA buffer (150 mM NaCl, 5 mM $K_3[FeCN_6]$, 5 mM $K_4[FeCN_6]$, 2 mM $MgCl_2$, 40 mM sodium citrate-Na_2HPO_4, pH 6.0) for 16 hours at 37ºC (no CO_2).

Immunofluorescence

A-549 cells were seeded on coverslips, allowed to attach overnight and treated as indicated. α-tubulin: cells were fixed and permeabilized with methanol (20 minutes. -20°C), blocked with 1% BSA in PBS and incubated with mouse anti-α-tubulin antibody (1:300, Amersham N365, 60 minutes, 37°C) and antimouse DyLight-488 conjugated donkey antibody (1:100, Pierce SA5-10166, 60 minutes, 37°C). F-actin: was stained with 0.5 μg/mL TRITC conjugated phalloidin (SigmaAldrich P1951, 20 minutes, room temperature) in PBS in cells fixed with 4% formaldehyde (10 minutes, 4°C) and permeabilized with 0.5% Triton X-100 (10 minutes, room temperature). DNA was counterstained with 0.1 μg/mL DAPI (10 minutes, room temperature), coverslips were mounted in 25 mg/mL 1,4-diazabicyclo [2, 2,

2]octane (DABCO) in 50% glycerol in PBS and sealed with nail polish. Images were acquired under fluorescence microscope using identical settings for every sample. Actin polymerization assay Experiment was carried out with Actin polymerization kit (Cytoskeleton #BK003) according to manufacturer's instructions. Briefly, 0.4 mg/mL pyrenelabelled rabbit muscle G-actin was suspended at in 0.2 mM $CaCl_2$, 5 mM Tris-HCl, pH 8.0, allowed to depolymerize (1 hour, 4°C) and cleared by centrifugation (16,100 x g, 30 minutes, 4°C). Tested compounds diluted in DMSO (final concentration 1%) were added to G-actin solution and baseline fluorescence was determined (20 minutes, RT). Polymerization was initiated by the addition of 50 mM KCl, 2 mM $MgCl_2$, 5 mM guanidine carbonate, 1 mM ATP, 10 mM Tris-HCl, pH 7.5 (final concentrations) and allowed to proceed for 1 hour. Data was gathered using Tecan Infinite M1000PRO in kinetic mode. Fluorescence was read using excitation wavelength set to 350 ± 20 nm and emission wavelength to 410 ± 10 nm every 30 seconds from the moment of compound addition until the signal reached plateau.

Cytometry

A-549 cells were seeded in Petri dishes, allowed to attach overnight and treated as indicated. Drug-treated cells were harvested, fixed with 70% ethanol (overnight or longer, -20°C), rehydrated in PBS, stained with 20 μg/mL propidium iodide and 100 μg/mL RNase A in PBS (1 hour, room temperature) and analyzed directly. To detect DNA synthesis, cells were labelled with 20 μM 5-bromo-2'-deoxyuridine (BrdU) for 1 hour before treatment end and fixed in 70% ethanol (overnight or longer, -20°C). Following brief rehydration with PBS (10 minutes on ice), DNA was denatured with 2 M HCl (30 minutes, room temperature) and suspension was neutralized with 0.1 M sodium tetraborate, pH 8.5 (10 minutes, room temperature). Cells were blocked with 1% BSA in PBS (15 minutes, room temperature), incubated with mouse anti-BrdU antibody (1:100, CalBioChem #NA61, 1 hour, 37°C), and incubated with anti-mouse FITC-conjugated donkey antibody (1:200, Jackson 715-095-151, 30 minutes, 37°C). To detect mitotic cells, ethanol-fixed cells were rehydrated with PBS (10 minutes on ice), blocked with 1% BSA in PBS and incubated with mouse anti-MPM2 antibody (1:100, DAKO M3514, 1 hour, 37°C), and antimouse FITC-conjugated donkey antibody (1:100, Jackson 715-095-151, 45 minutes, 37°C). To detect DNA damage, ethanol-fixed cells were rehydrated with PBS (10 minutes on ice), washed three times with 1% BSA in PBS and incubated with Alexa488-conjugated mouse anti-γH2AX antibody (1:40, BioLegend #613406, 1 hour, 37°C). For BrdU, MPM-2 and γH2AX, DNA was counterstained with 5 μg/μL propidium iodide and 100 μg/μL RNaseA in PBS (30 minutes, RT) and cells were processed by flow cytometry. 10 $x10^3$ cells were scored for each assay.

Supplementary References SR:

1. Borowski, E.; Barbara, S.; Maria, D.; Cybulski, M.; Szelejewski, W.; Obukowicz, J.; Bontemps-Gracz, M.; Wysocka, M.; Mazerski, J.; Punda, P.; et al. Asymmetrically Substituted Anthrapyridazone Derivatives as Cytostatics. U.S. Patent No 9,096,536, 4 August 2015.
2. Pawłowska, J.; Tarasiuk, J.; Wolf, C.R.; Paine, M.J.I.; Borowski, E. Differential ability of cytostatics from anthraquinone group to generate free radicals in three enzymatic systems: NADH dehydrogenase, NADPH cytochrome P450 reductase, and xanthine oxidase. *Oncol. Res.* **2003**, *13*, 245–252.
3. Ratajczak, M.Z.; Zuba-Surma, E.; Wojakowski, W.; Suszynska, M.; Mierzejewska, K.; Liu, R.; Ratajczak, J.; Shin, D.M.; Kucia, M. Very small embryonic-like stem cells (VSELs) represent a real challenge in stem cell biology: Recent pros and cons in the midst of a lively debate. *Leukemia* **2014**, *28*, 473–484, doi:10.1038/leu.2013.255.
4. Danova-Alt, R.; Heider, A.; Egger, D.; Cross, M.; Alt, R. Very small embryonic-like stem cells purified from umbilical cord blood lack stem cell characteristics. *PLoS ONE* **2012**, *7*, 1–11, doi:10.1371/journal.pone.0034899.
5. Laverdière, I.; Boileau, M.; Neumann, A.L.; Frison, H.; Mitchell, A.; Ng, S.W.K.; Wang, J.C.Y.; Minden, M.D.; Eppert, K. Leukemic stem cell signatures identify novel therapeutics targeting acute myeloid leukemia. *Blood Cancer J.* **2018**, *8*, doi:10.1038/s41408-018-0087-2.
6. Nishino, T.; Osawa, M.; Iwama, A. New approaches to expand hematopoietic stem and progenitor cells. *Expert Opin. Biol. Ther.* **2012**, *12*, 743–756.
7. Lu, R.; Miao, D. Very small embryonic like (VSEL) stem cells. *J. Nanjing Med. Univ.* **2008**, *22*, 265–268, doi:10.1016/s1007-4376(08)60077-5.
8. Kuruca, S.E.; Çelik, D.D.; Özerkan, D.; Erdemir, G. Characterization and isolation of very small embryonic-like (VSEL) Stem cells obtained from various human hematopoietic cell sources. *Stem Cell Rev. Rep.* **2019**, *15*, 730–742, doi:10.1007/s12015-019-09896-1.

9. Sovalat, H.; Scrofani, M.; Eidenschenk, A.; Hénon, P. Human very small embryonic-like stem cells are present in normal peripheral blood of young, middle-aged, and aged subjects. *Stem Cells Int.* **2016**, *2016*, doi:10.1155/2016/7651645.
10. Kurosawa, S.; Iwama, A. Aging and leukemic evolution of hematopoietic stem cells under various stress conditions. *Inflamm. Regen.* **2020**, *40*, 29.
11. Hanekamp, D.; Cloos, J.; Schuurhuis, G.J. Leukemic stem cells: Identification and clinical application. *Int. J. Hematol.* **2017**, *105*, 549–557, doi:10.1007/s12185-017-2221-5.
12. Madhumathi, J.; Sridevi, S.; Verma, R.S. CD25 targeted therapy of chemotherapy resistant leukemic stem cells using DR5 specific TRAIL peptide. *Stem Cell Res.* **2017**, *19*, 65–75, doi:10.1016/j.scr.2017.01.001.
13. Kräter, M.; Jacobi, A.; Otto, O.; Tietze, S.; Müller, K.; Poitz, D.M.; Palm, S.; Zinna, V.M.; Biehain, U.; Wobus, M.; et al. Bone marrow niche-mimetics modulate HSPC function via integrin signaling. *Sci. Rep.* **2017**, *7*, 1–15, doi:10.1038/s41598-017-02352-5.
14. Birnie, G.D. The HL60 cell line: A model system for studying human myeloid cell differentiation. *Br. J. Cancer. Suppl.* **1988**, *9*, 41–45.
15. Collins, S.J.; Gallo, R.C.; Gallagher, R.E. Continuous growth and differentiation of human myeloid leukaemic cells in suspension culture. *Nature* **1977**, *270*, 347–349.
16. Gallagher, R.; Collins, S.; Trujillo, J.; McCredie, K.; Ahearn, M.; Tsai, S.; Metzgar, R.; Aulakh, G.; Ting, R.; Ruscetti, F.; et al. Characterization of the continuous, differentiating myeloid cell line (HL-60) from a patient with acute promyelocytic leukemia. *Blood* **1979**, *54*, 713–733.
17. Collins, S.J.; Ruscetti, F.W.; Gallagher, R.E.; Gallo, R.C. Normal functional characteristics of cultured human promyelocytic leukemia cells (HL-60) after induction of differentiation by dimethylsulfoxide. *J. Exp. Med.* **1979**, *149*, 969–974, doi:10.1084/jem.149.4.969.
18. Collins, S.J.; Ruscetti, F.W.; Gallagher, R.E.; Gallo, R.C. Terminal differentiation of human promyelocytic leukemia cells induced by dimethyl sulfoxide and other polar compounds. *Proc. Natl. Acad. Sci. USA* **1978**, *75*, 2458–2462, doi:10.1073/pnas.75.5.2458.
19. Gieseler, F.; Bauer, E.; Nuessler, V.; Clark, M.; Valsamas, S. Molecular effects of topoisomerase II inhibitors in AML cell lines: Correlation of apoptosis with topoisomerase II activity but not with DNA damage. *Leukemia* **1999**, *13*, 1859–1863, doi:10.1038/sj.leu.2401570.
20. Chikamori, K.; Hill, J.E.; Grabowski, D.R.; Zarkhin, E.; Grozav, A.G.; Vaziri, S.A.J.; Wang, J.; Gudkov, A.V.; Rybicki, L.R.; Bukowski, R.M.; et al. Downregulation of topoisomerase IIβ in myeloid leukemia cell lines leads to activation of apoptosis following all-trans retinoic acid-induced differentiation/growth arrest. *Leukemia* **2006**, *20*, 1809–1818, doi:10.1038/sj.leu.2404351.
21. Wangen, R.; Aasebø, E.; Trentani, A.; Døskeland, S. O.; Bruserud, Ø.; Selheim, F.; Hernandez-Valladares, M. Preservation Method and Phosphate Buffered Saline Washing Affect the Acute Myeloid Leukemia Proteome. *International journal of molecular sciences*, **2018**, 19, 296. https://doi.org/10.3390/ijms19010296
22. Fricker, S.P. The application of sulforhodamine B as a colorimetric endpoint in a cytotoxicity assay. *Toxicol. Vitr.* **1994**, *8*, 821–822, doi:10.1016/0887-2333(94)90076-0.

Original, uncropped, unadjusted pictures

Western blots

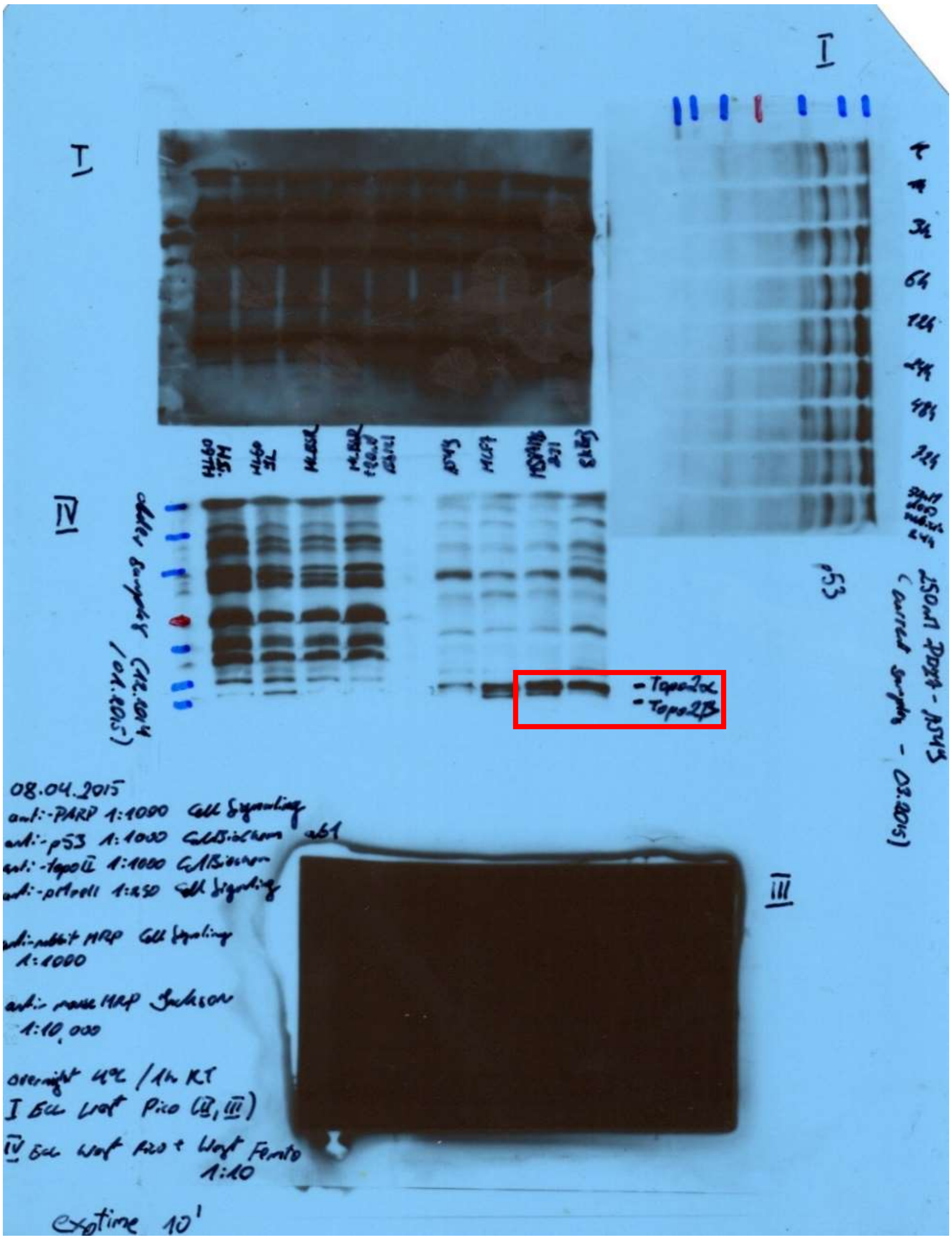

Please refer to Figure 2C for the details. Region of interest is marked with red rectangle.

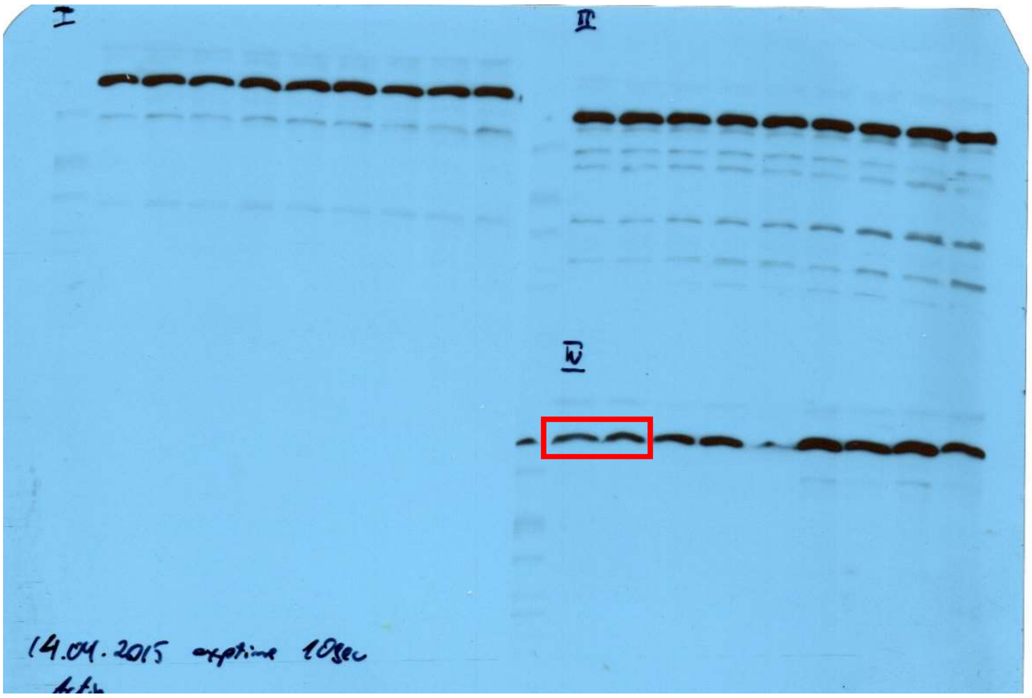

Please refer to Figure 2C for the details. Region of interest is marked with red rectangle.

Agarose gels

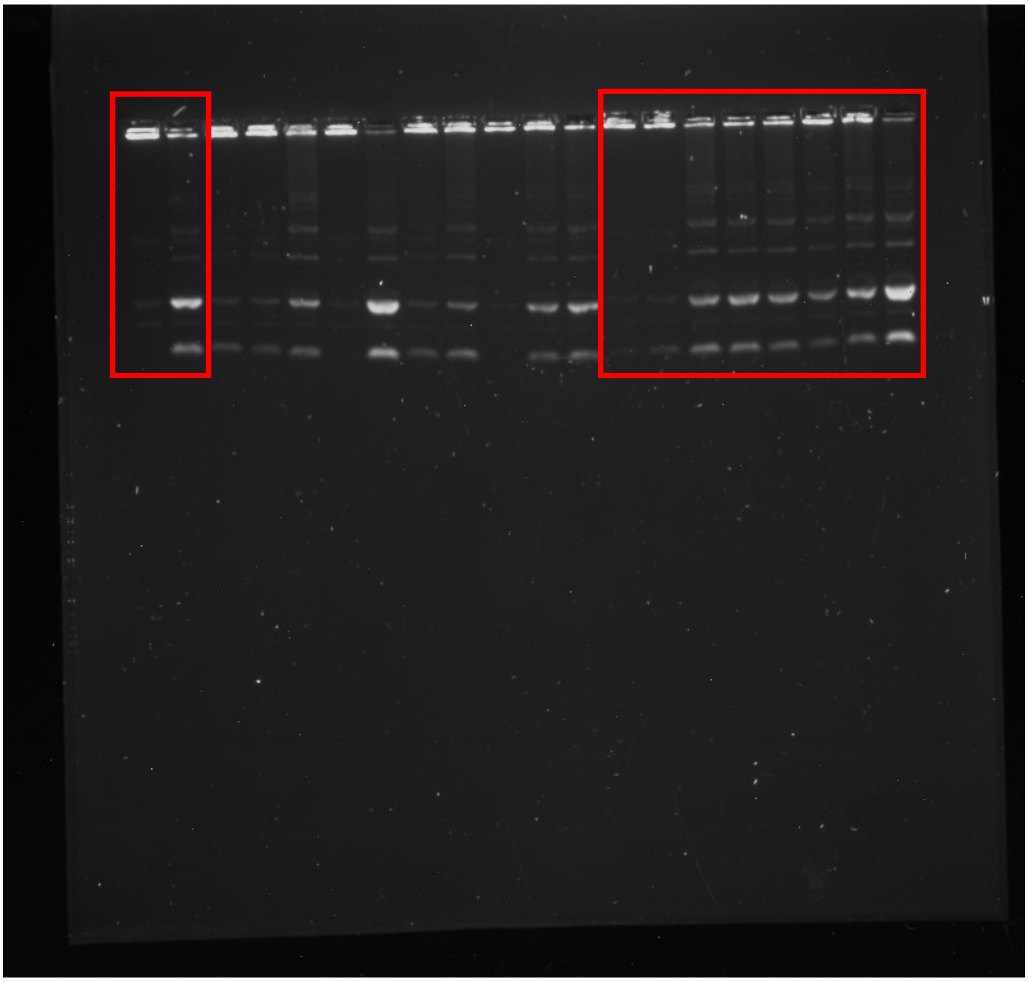

Please refer to Figure 4A for the details. Region of interest is marked with red rectangles.
